# Influence of spatial camera resolution in high-speed videoendoscopy on laryngeal parameters

**DOI:** 10.1371/journal.pone.0215168

**Published:** 2019-04-22

**Authors:** Patrick Schlegel, Melda Kunduk, Michael Stingl, Marion Semmler, Michael Döllinger, Christopher Bohr, Anne Schützenberger

**Affiliations:** 1 Dep. of Otorhinolaryngology, Div. of Phoniatrics and Pediatric Audiology, University Hospital Erlangen, Friedrich-Alexander-University Erlangen-Nürnberg, Erlangen, Germany; 2 Dep. of Communication Sciences and Disorders, Louisiana State University, Baton Rouge, LA, United States of America; 3 Department Mathematics, Applied Mathematics II, FAU Erlangen-Nürnberg, Erlangen, Germany; 4 Dep. of Otorhinolaryngology, University Hospital Regensburg, Regensburg, Germany; Griffith University, AUSTRALIA

## Abstract

In laryngeal high-speed videoendoscopy (HSV) the area between the vibrating vocal folds during phonation is of interest, being referred to as glottal area waveform (GAW). Varying camera resolution may influence parameters computed on the GAW and hence hinder the comparability between examinations. This study investigates the influence of spatial camera resolution on quantitative vocal fold vibratory function parameters obtained from the GAW. In total 40 HSV recordings during sustained phonation (20 healthy males and 20 healthy females) were investigated. A clinically used Photron Fastcam MC2 camera with a frame rate of 4000 fps and a spatial resolution of 512×256 pixels was applied. This initial resolution was reduced by pixel averaging to (1) a resolution of 256×128 and (2) to a resolution of 128×64 pixels, yielding three sets of recordings. The GAW was extracted and in total 50 vocal fold vibratory parameters representing different features of the GAW were computed. Statistical analyses using SPSS Statistics, version 21, was performed. 15 Parameters showing strong mathematical dependencies with other parameters were excluded from the main analysis but are given in the Supporting Information. Data analysis revealed clear influence of spatial resolution on GAW parameters. Fundamental period measures and period perturbation measures were the least affected. Amplitude perturbation measures and mechanical measures were most strongly influenced. Most glottal dynamic characteristics and symmetry measures deviated significantly. Most energy perturbation measures changed significantly in males but were mostly unaffected in females. In females 18 of 35 remaining parameters (51%) and in males 22 parameters (63%) changed significantly between spatial resolutions. This work represents the first step in studying the impact of video resolution on quantitative HSV parameters. Clear influences of spatial camera resolution on computed parameters were found. The study results suggest avoiding the use of the most strongly affected parameters. Further, the use of cameras with high resolution is recommended to analyze GAW measures in HSV data.

## Introduction

Regardless of the area of research, small factors can often exert a strong influence on the results obtained and the parameters calculated. For example, in mathematical modeling a small change of only one parameter can have a large effect on other parts of the model [1, 2]. In spectroscopy a small difference in a measured spectrum can be a crucial factor for determining the exact kind of material the analyzed light was emitted from [3] and in medicine slightly different symptoms can indicate completely different diseases [4]. Equally, in laryngeal high-speed video endoscopy (HSV) different factors can significantly influence the outcome of a measurement obscuring the distinction between normal and pathological laryngeal dynamics.

During phonation, the vocal folds are set in motion by a stream of air rising from the lungs. This results in a periodic oscillation of the vocal folds cyclically interrupting the airflow in the process producing audible sound [5, 6]. Different ranges of the frequency of vocal fold oscillation are given in literature with upper boundaries ranging from about 250 Hz [7, 8] to 400 Hz [9] in females. However, in some studies upper boundaries of 500 Hz [10] or even 1000 Hz [11] are presented. Still the vocal folds oscillation frequency can exceed easily 1000 Hz during singing [12]. While the vocal folds are the vibratory sound source [13], the sound is further modulated by tongue and lips finally generating the audible voice and speech [5, 6].

A healthy voice is usually associated with periodic, symmetric vocal fold oscillation and glottal closure [14–16]; although a recent investigation of the three dimensional vibration characteristics of the vocal folds implies that this idea of healthy vocal fold vibration may be a bit too simplistic in regard of vertical vibration components [17]. A pathological voice, also in absence of structural or neurologic impairments, is respectively usually associated with irregular and asymmetric vibrations of the vocal folds [18–20]. HSV can be used to examine healthy and disordered voices and is a powerful method for examining the phonation process by recording the fast vibrations of the vocal folds [21–24].

**Fig 1:**
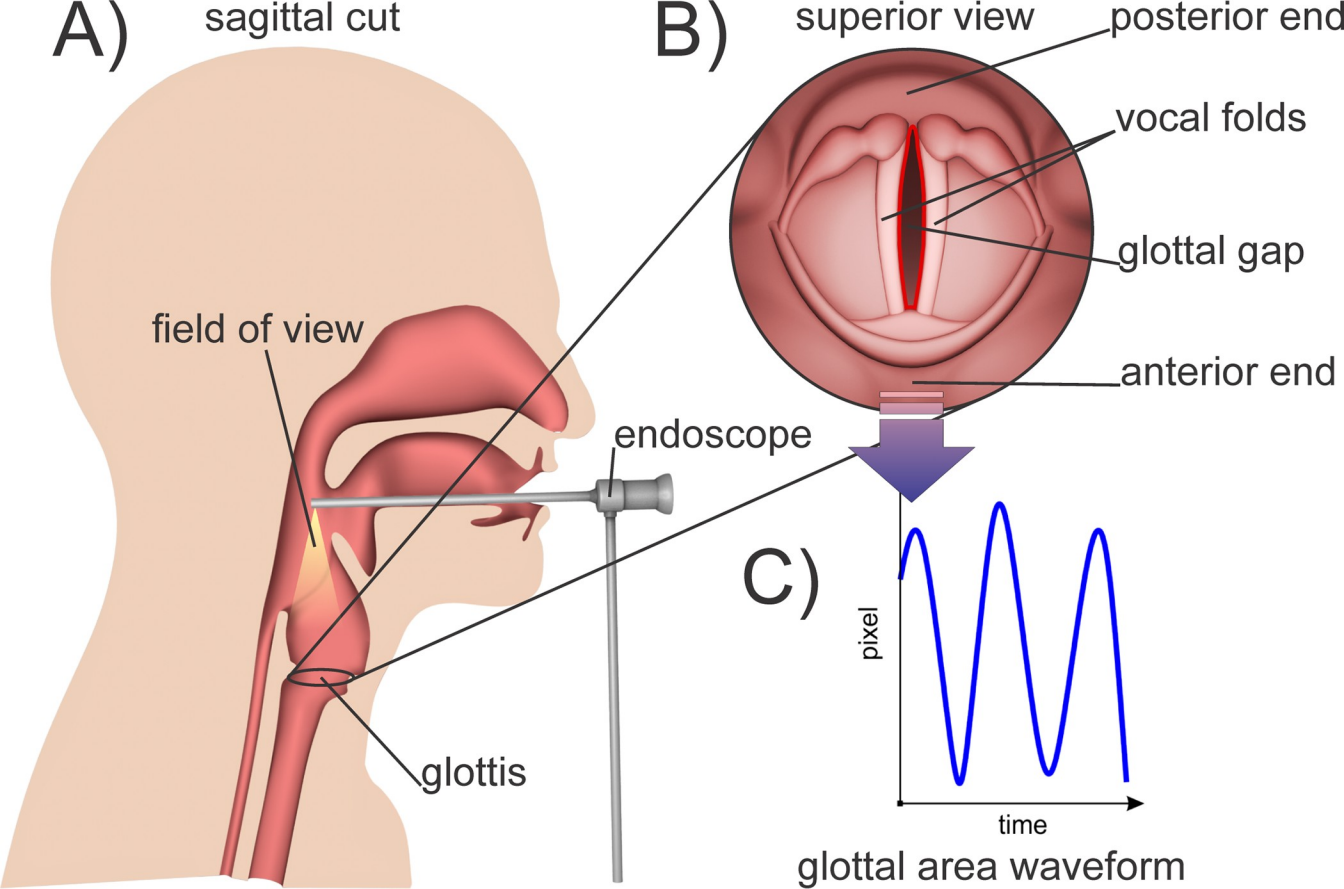
A) Recording of the vocal fold oscillations via a rigid endoscope being attached to a high-speed camera. B) Superior view of the vocal folds as seen with the endoscope. C) Computed GAW: number of pixels enclosed within the glottis over time.

In Fig 1 a typical clinical examination situation for HSV is shown. The vibrations of the vocal folds are recorded applying a rigid endoscope [25]. From the recorded images of the vocal folds, the area in between the vocal folds, the “glottal area”, is calculated. Subsequently the area from each consecutively recorded image is composited to the function of the glottal area over time, the glottal area waveform (GAW) [26]. Some slightly different definitions of the GAW exist [27–30]. In this work, the GAW is defined as the function of the glottal area in pixels over frames. This definition was used for the calculation of all parameters in this work.

Parameters calculated directly from obtained data grant a certain objectivity of the analysis in contrast to subjective evaluation of voice quality and laryngeal imaging, which often results in a low inter- and intra-rater reliability [31, 32]. Still, different influences on parameters obtained from HSV and the audio signal, being sometimes recorded parallely to HSV [33, 34], are not understood in detail and are currently under investigation [35–40]. There are several potential factors that can affect parameter values. These factors include age and gender of the subjects [35]. Also more controllable factors like the recording frame rate [36, 37], the analyzed interval length [38–40] and, as this work aims to prove that the spatial image resolution play a role. The influence of the recording frame rate was already investigated to some extent for GAW parameters and it was found that 90% of all 20 investigated parameters were affected by changes in the frame rate. Since the parameter changes between 4000 and 15,000 frames per second (fps) were relatively small for glottal dynamic characteristics, glottal periodicity and perturbation characteristics, the application of recoding frame rates of 4000 fps in clinical studies was judged as justified. Furthermore, the determination of normative parameter values based on recording frame rates was suggested [36]. Also acoustic measures were investigated and a minimal sampling frequency of 26 kHz was suggested to avoid introducing errors due to a too low sampling frequency [37]. Studies examining the interval length in view of minimal number of oscillation cycles and comparability of perturbation measures in acoustic signals exist [38, 39]. In one of these studies, a minimum sequence length in the order of 100 cycles for the calculation of stable perturbation measures was suggested [38]. In the other one, it was found that frequency and amplitude perturbation were not in agreement for different analysis systems, although 110 cycles were used for calculation [39]. Besides the audio signal, another type of signal for examining the vocal fold vibrations similar to the GAW, the electroglottographic (EGG) signal, was investigated in view of different sequence lengths. It was found that two of nine perturbation parameters for the audio signal and for the EGG signal (although not the same parameters) were affected by changing sequence lengths [40]. It is known that the accuracy of approaches dealing with a quantitative analysis of the vocal fold vibration depends on spatial chip or camera resolution [41] and from other fields of research it is also known, that image resolution can have a major influence on the results of analysis [42–44]. However, no systematic studies on the influence of spatial HSV resolution on computed and quantitative GAW parameters have been performed yet.

To fill this gap, we investigate the influence of changing spatial resolution on 50 different potential GAW parameters. This work aims to show the necessity of considering possibly deviating camera resolutions, when comparing different studies. Various parameters are discussed in regard of the effect of changing spatial resolution. Parameters that are strongly influenced by changing spatial resolution can be used only to a limited extent or not at all for distinguishing healthy and disordered subjects in clinics. Hence those strongly affected parameters should be excluded from further studies, reducing the large number of parameters used in voice analysis. Summarizing, the aims of this work are (1) to investigate the influence of changing image resolution on GAW parameters and (2) to find parameters that are comparatively unaffected by changing resolution. By statistically analyzing and discussing the changes of the parameters, these aims were achieved. Preliminary results from this study were presented at the 11^th^ International Conference on Voice Physiology and Biomechanics (ICVBP) [45].

## Methods

40 HSV recordings of 20 healthy male and 20 healthy female subjects were investigated. The age of the female subjects varied from 17 to 29 years (average 19.9 years) and from 22 to 29 (average age 24.2) for males. The recordings were taken endoscopically from subjects during sustained phonation of the vowel /i/ at a comfortable pitch and loudness level. All recordings were chosen from our existing clinical database and all had a length of at least 250 milliseconds (ms). The study was approved by the ethic committee of the Medical School at Friedrich-Alexander-University Erlangen-Nürnberg (no. 290_13B). Written consent was obtained by the subjects or, in case of underage, by their legal guardians.

**Fig 2:**
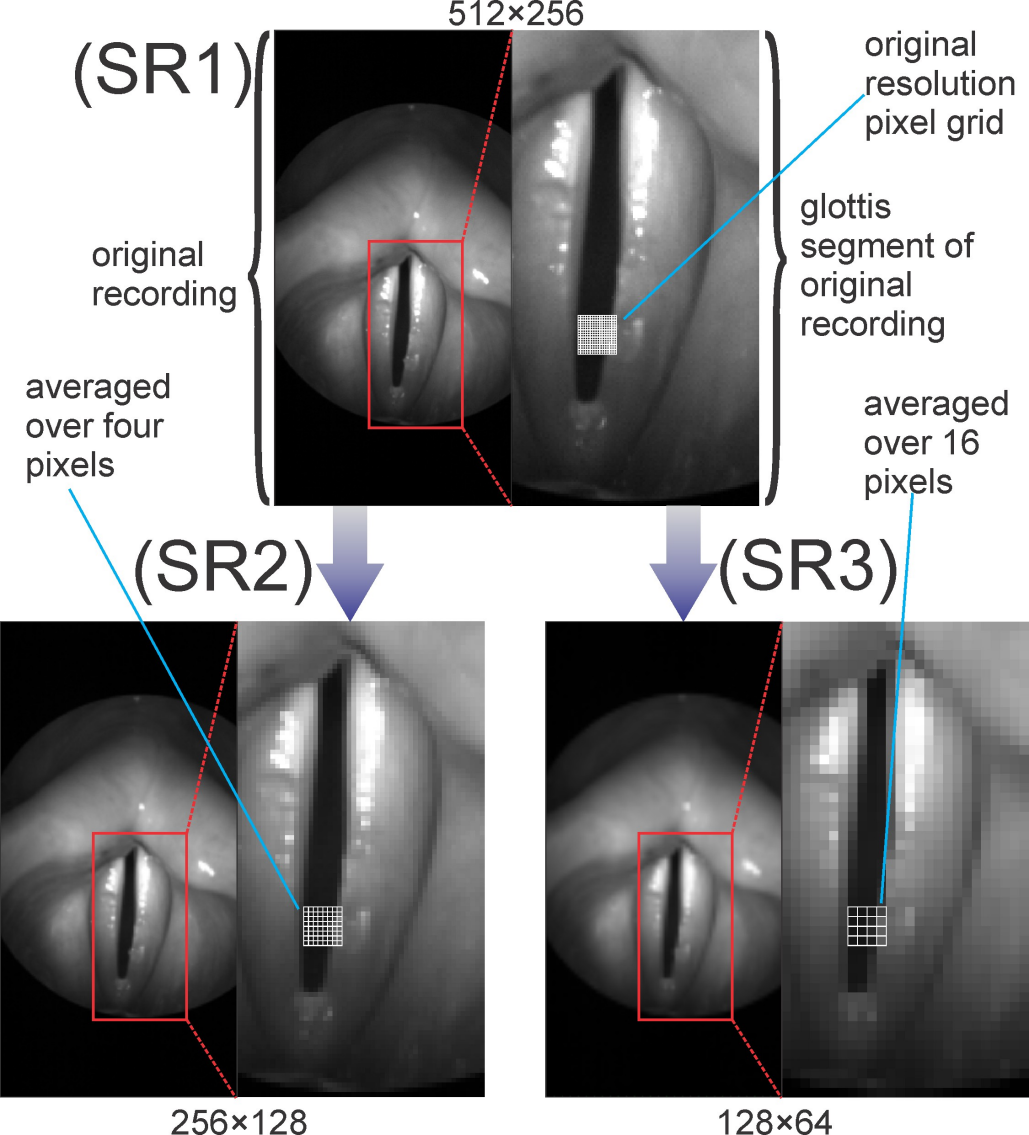
Reduction of image resolution by pixel averaging and selection of a rectangular subsection.

In this study videos were included showing (1) good recording quality and (2) visibility of the entire glottis. The recording quality of all videos was previously judged by a subjective rater on a 1 to 6 scale with 1 being the best rating. Included in this study were only videos with a rating of at least 3, meaning good brightness so that all details in the recording were clearly visible and good contrast i.e. sharp glottis edges. All videos were recorded by a clinically used Photron Fastcam MC2 camera with a frame rate of 4000 fps and a spatial resolution of 512×256 pixels (SR1). The resolution of these 40 initial recordings was then reduced to a resolution of 256×128 pixels (SR2) and to a resolution of 128×64 pixels (SR3) yielding two new sets of data. As depicted in Fig 2, both of the reduced sets were calculated from the original set by averaging over the intensity values of squares of 2×2 (SR2) and 4×4 (SR3) pixels resulting in a mean intensity value for each new pixel, which was rounded to the nearest integer number. The apparent low initial resolution was due to the common clinical settings; since currently used clinical high-speed cameras do not have very high resolutions [46–49].

### Segmentation

After creating the two new datasets with reduced resolution from each original recording, for further analysis, a rectangular subsection of the recording containing the entire glottal area was selected (Fig 2). Only these subsections of the recordings were used for segmentation to exclude the larger insignificant parts of the video from analysis. Each original recording and its two reduced versions form a “triad” resulting in 20 female and 20 male recording triads.

**Fig 3:**
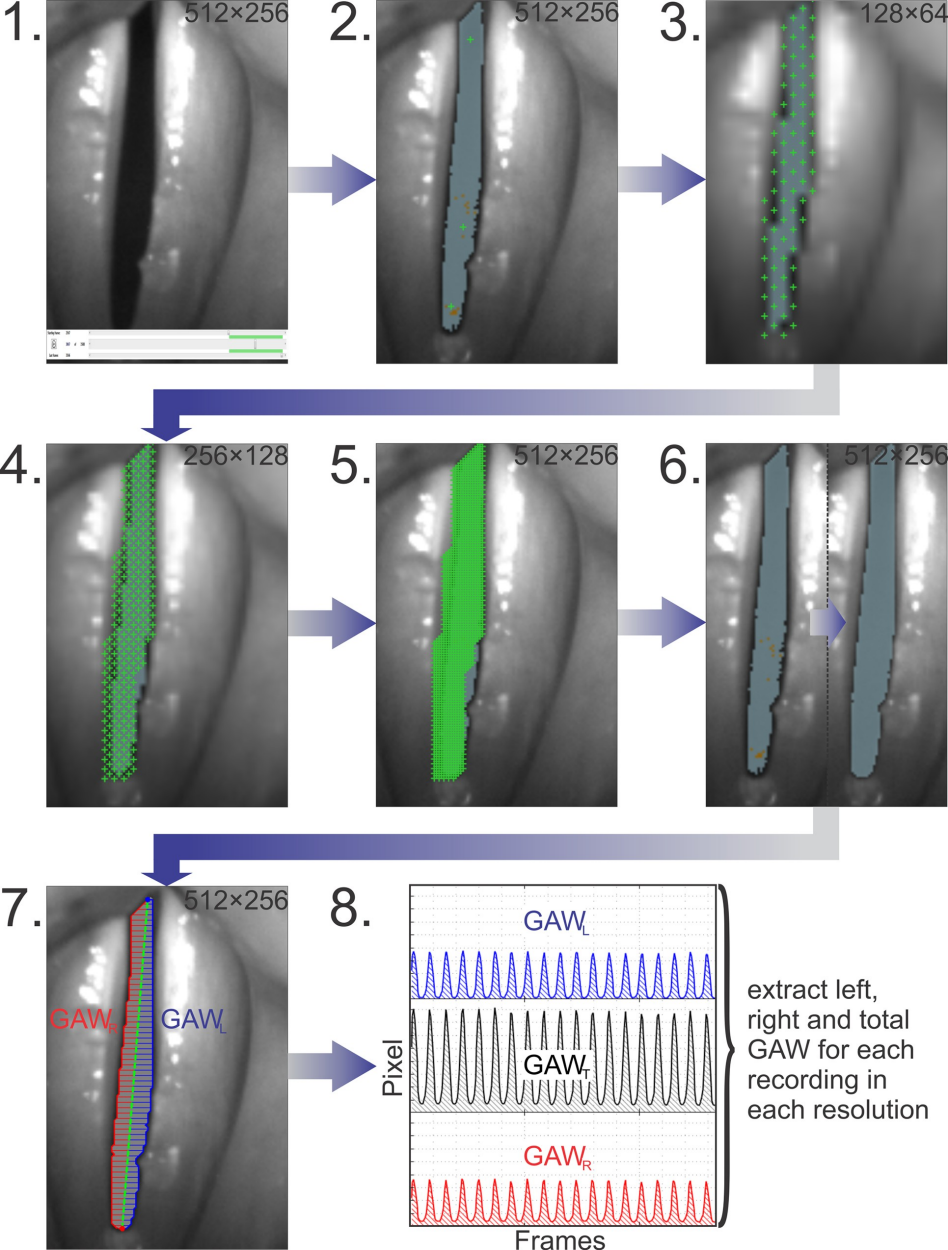
Scheme of the segmentation process: 1. Selection of a 1000 frames section, 2. Rough pre-segmentation, 3. Initial grid in the lowest resolution data, 4. First condensing of the grid, 5. Second condensing of the grid, 6. Adjustment of brightness thresholds, 7. Calculation of the partial GAWs, and 8. Extraction of all GAWs.

The recorded triads were segmented using an in house developed version of Glottis Analysis Tools (GAT– 2018). The applied segmentation procedure is illustrated for each step in Fig 3. For illustrative reasons, in Fig 3.1 –Fig 3.7 only single frames of the video are shown. However, the segmentation was carried out considering all 1000 frames resulting in the GAW of which a section is shown in Fig 3.8. The procedure for each triad was as follows:

1 A suitable segment of 1000 frames (250 ms) in the original recording was selected. A segment was considered suitable if the glottis was completely visible, vibrated regularly and the field of view moved as little as possible in lateral and horizontal direction on the recording.
2 For these 1000 frames in the original recording, a rough pre-segmentation procedure was applied, placing first seed points and preadjusting the brightness thresholds. Each seed point marks one initial position for segmentation. Starting from each seed point position all pixels are added to the glottal area that (1) have a lower brightness value than the chosen brightness threshold and (2) are neighboring the seed point or a pixel that already was added to the glottal area. The use of the seed points as a grid ensured a high degree of objective segmentation, as this avoided setting the seed points on specific, subjective positions. Moreover a rather close-meshed grid ensured a minimal error by not segmenting the dark areas within the glottis.
3–6 These preadjusted settings were then exported to the other recordings of the triad. For the lowest resolution the existing seed points were exchanged for a regular mesh of seed points that was condensed by a factor of two and four for the higher resolutions. The mesh was created semi-automatically by using a seed point drawing tool. Subsequently the previously preadjusted brightness thresholds were finalized.
7 After segmentation, the contour of the glottal area was calculated as described in [50] and the total glottal area was subdivided into two sides. From each side, the left (GAW_L_) and right (GAW_R_) partial glottal area waveforms were computed by numerical integration over the distances between the midline and the left and right contour lines.
8 For all three resolutions, the total (GAW_T_), left (GAW_L_) and right (GAW_R_) glottal area waveforms were extracted.

### Parameter computation

Overall, 120 GAW_T_ signals plus 120 GAW_L_ and GAW_R_ signals were calculated. As S1 Fig illustrates, in a first step, the cycles of GAW_T_ and partial GAWs were determined. Maximum based cycle detection was used, this means each cycle starts at a sufficiently distinct local maximum and ends before the next significant local maximum. Starting with the third detected cycle for each signal, 20 consecutive cycles were selected. For these cycles, for each GAW_T_, 41 different cycle based parameters were computed. For each pair of one GAW_L_ and the corresponding GAW_R_, nine cycle based symmetry parameters were computed, yielding in total 50 different parameters. Parameters were averaged over all 20 cycles. The number of cycles was not chosen too large in order to keep the influencing factors that change over the course of the recording low; factors such as frequency shifts in the phonation or field of view movements that can influence parameter calculation [51].

All investigated parameters are summarized with a brief description and corresponding references [52–70] under S1 Table in the section “supporting information”. Parameters that were later excluded from the analysis are marked. The parameters were divided into seven groups based on the dynamical characteristics they describe:

1. The group “**fundamental period measures**” (FPM) contains parameters that are related to the perceived pitch of the voice [71].
2. Parameters describing changes in GAW cycle lengths were grouped under “**period perturbation measures**” (PPM). They are associated with the stability of the phonatory system, the larger the parameter value (with the exception of *Time Periodicity* (*TP*), which decreases with increasing perturbation), the larger the changes in cycle lengths. Since no voice is perfectly free of perturbation, they usually do not reach their optimal value that indicates no perturbation, still they are expected to reach higher, or in case of *TP* lower, values for more erratically oscillating vocal folds [72].
3. Accordingly, eight parameters describing changes in the “dynamic range” (maximal area minus minimal area within one cycle) within one GAW were grouped as “**amplitude perturbation measures**” (APM). Similar to PPM, high values of amplitude perturbation are associated with disordered voices with the exception of *Amplitude Periodicity* (*AP*) for which, lower values indicate increased perturbation. The considered shimmer measures are associated with hoarseness when computed in acoustic signals, whereby this connection is not undisputed and the reliability of shimmer and jitter as independent indices of perceived vocal quality is questioned [73, 74].
4. Measures describing energy perturbation, respectively the difference in cycle energies within one GAW were grouped as “**energy perturbation measures**” (EPM). The cycle energy is calculated as the sum of squared sample values of all data points within the cycle. Compared to their counterparts in the APM, these measures describing energy perturbation are expected to be less susceptible to noise [75]. This is because they depend on multiple data points per cycle and not just on only one, as it is the case for their APM equivalents.
5. The measures calculated from the partial GAWs are summarized under the term “**symmetry measures**” (SM). They describe how similar the vibration of the left vocal fold is to the right one, and therefore, if there are differences in amplitude, frequency and other features of their oscillation characteristics. For healthy subjects, a comparatively higher symmetry of their vibration compared to unhealthy subjects is expected [14, 76].
6. Measures in the group “**glottal dynamic characteristics**” (GDC) are used to characterize ratios between different phases and changes in glottal area within one GAW cycle. Variations of GDC with changes in F0 and vocal intensity have been studied. Among others, it was found that the S*peed Quotient* varies directly with the vocal intensity [62]. Some GDC are associated with breathy voice or intensity and pressedness in voice, although only for calculations based on flow measures [77].
7. The “**mechanical measures**” (MM) mostly contain measures describing physical forces affecting the glottis and derivation measures. Since most of the, in this work, examined MM are measured in pixel-dependent units, they are expected to correlate strongly with the spatial video resolution. Some of the mechanical measures are given in “megapixel” (Mpx) to obtain values on a manageable scale.

### Statistical analysis

In the statistical analysis performed with SPSS Statistics, version 21, the parameters were compared between the three different HSV resolutions (SR1, SR2, SR3). Paired tests for connected samples were chosen for comparison. For all statistical tests, the H0 hypothesis was rejected if the p-value was below or equal to 0.05 (after Bonferroni correction for post hoc tests, see also S2 Fig). For the general linear model (GLM) repeated measures with 3 within-subject variables (for three different resolutions) for each test were chosen. A saturated model and the Type III sum of squares were used. The detailed workflow of the statistical analysis is depicted in S2 Fig.

## Results and discussion

**Fig 4:**
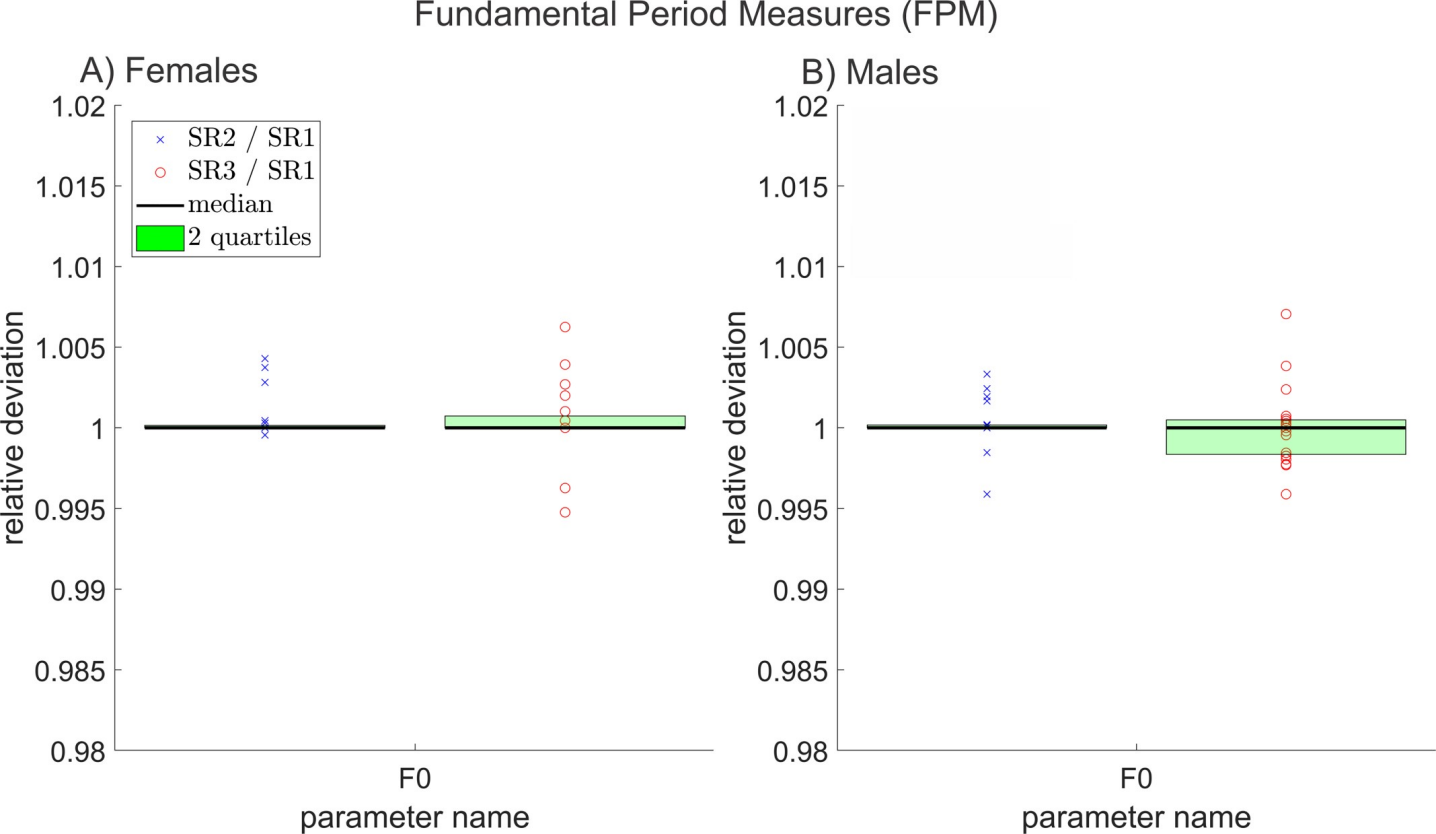
Relative deviations between all fundamental period measures, of reduced and original resolution data for A) females and B) males. (For some plots the green boxes are not visible because at least one half of the compared values did change only marginally or not at all between resolutions).

**Fig 5:**
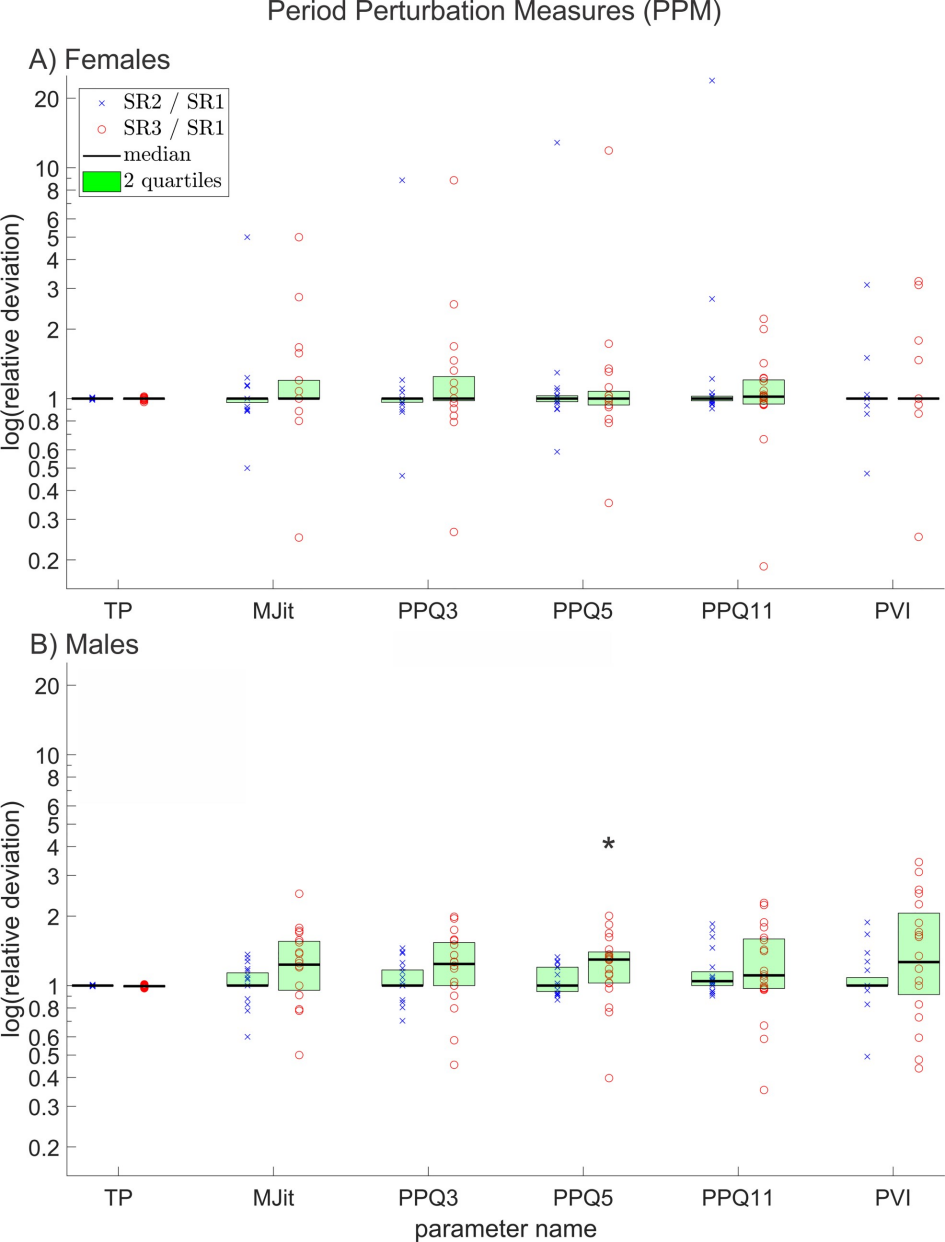
Relative deviations between all period perturbation measures, of reduced and original resolution data for A) females and B) males. The y-axis scaling is logarithmic. The * symbol indicates a statistically significant deviation (p≤0.05).

**Fig 6:**
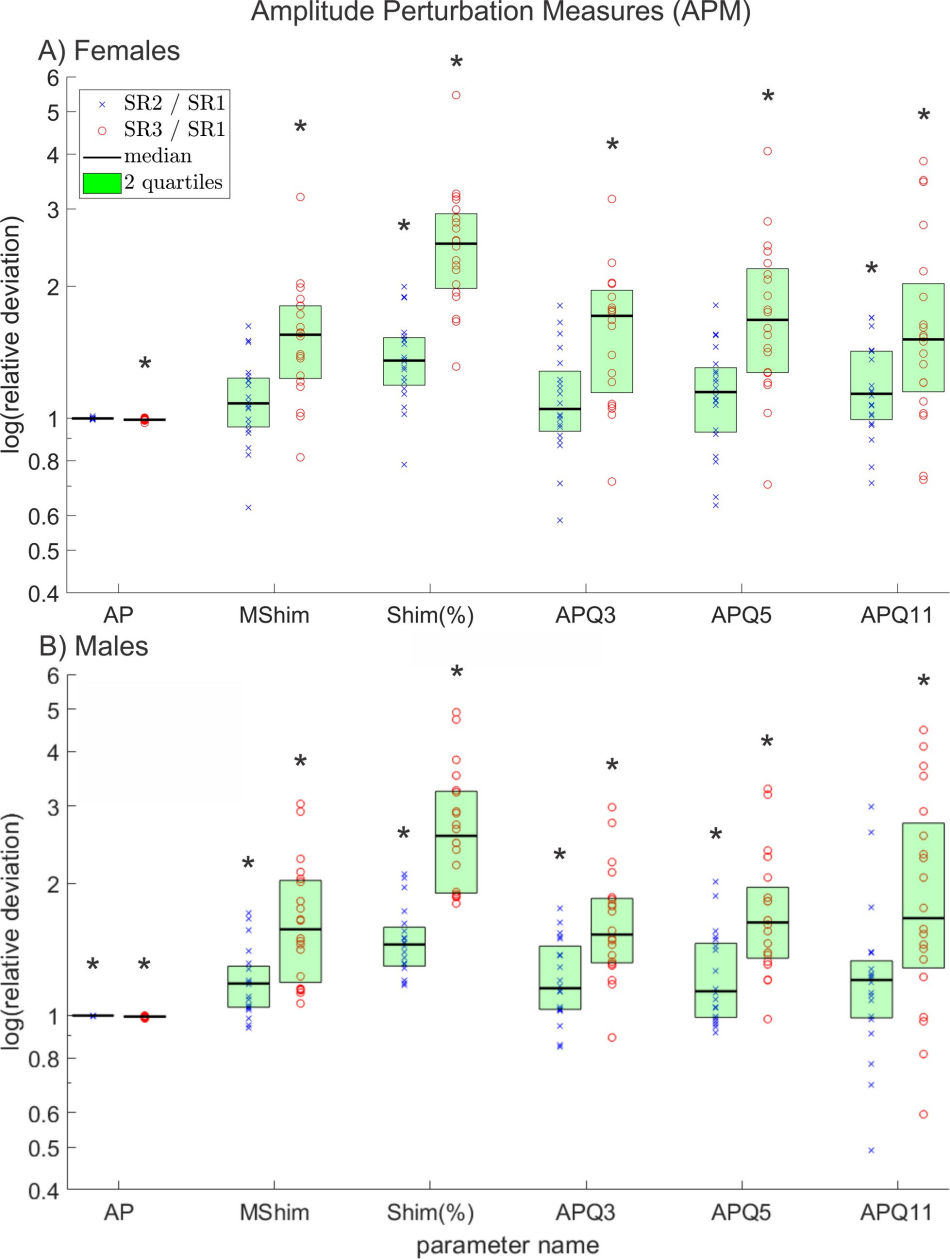
Relative deviations between all amplitude perturbation measures except AVI, of reduced and original resolution data for A) females and B) males. The y-axis scaling is logarithmic. The * symbols indicate statistically significant deviations (p≤0.05).

**Fig 7:**
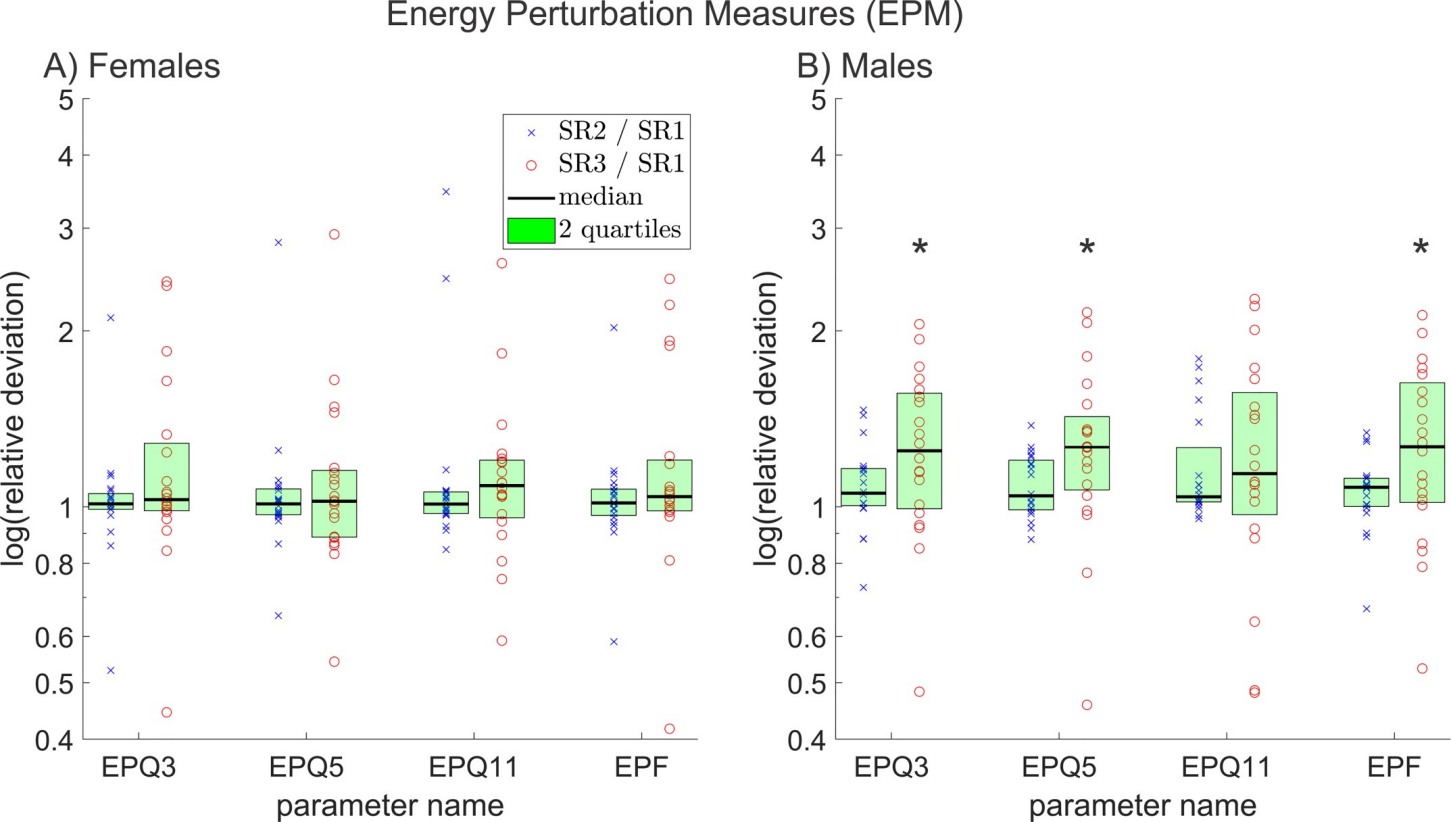
Relative deviations between all energy perturbation measures, of reduced and original resolution data for A) females and B) males. The y-axis scaling is logarithmic. The * symbols indicate statistically significant deviations (p≤0.05).

**Fig 8:**
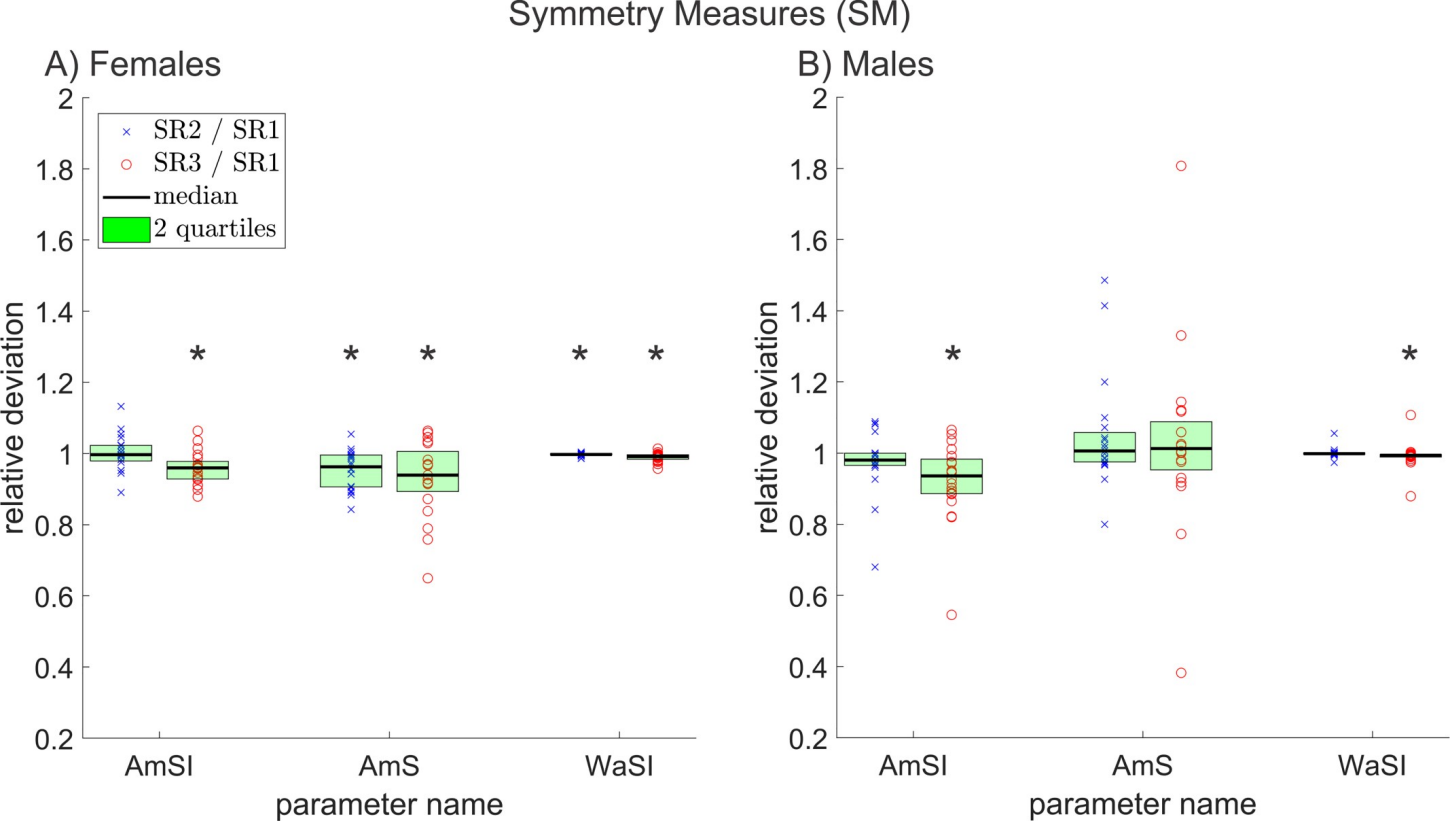
Relative deviations between all symmetry measures, of reduced and original resolution data for A) females and B) males. The * symbols indicate statistically significant deviations (p≤0.05).

**Fig 9:**
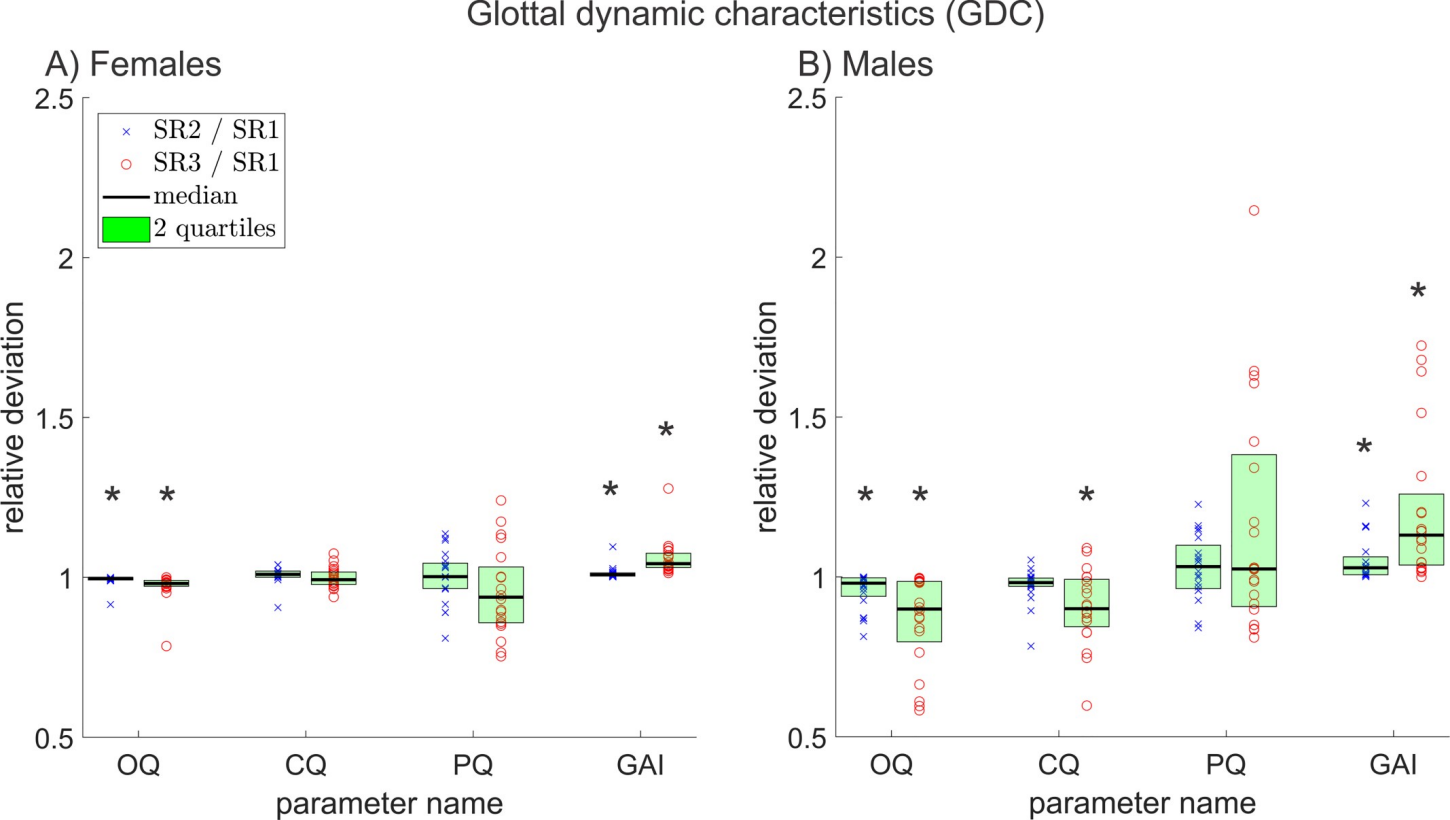
Relative deviations between all glottal dynamic characteristics, of reduced and original resolution data for A) females and B) males. The * symbols indicate statistically significant deviations (p≤0.05).

**Fig 10:**
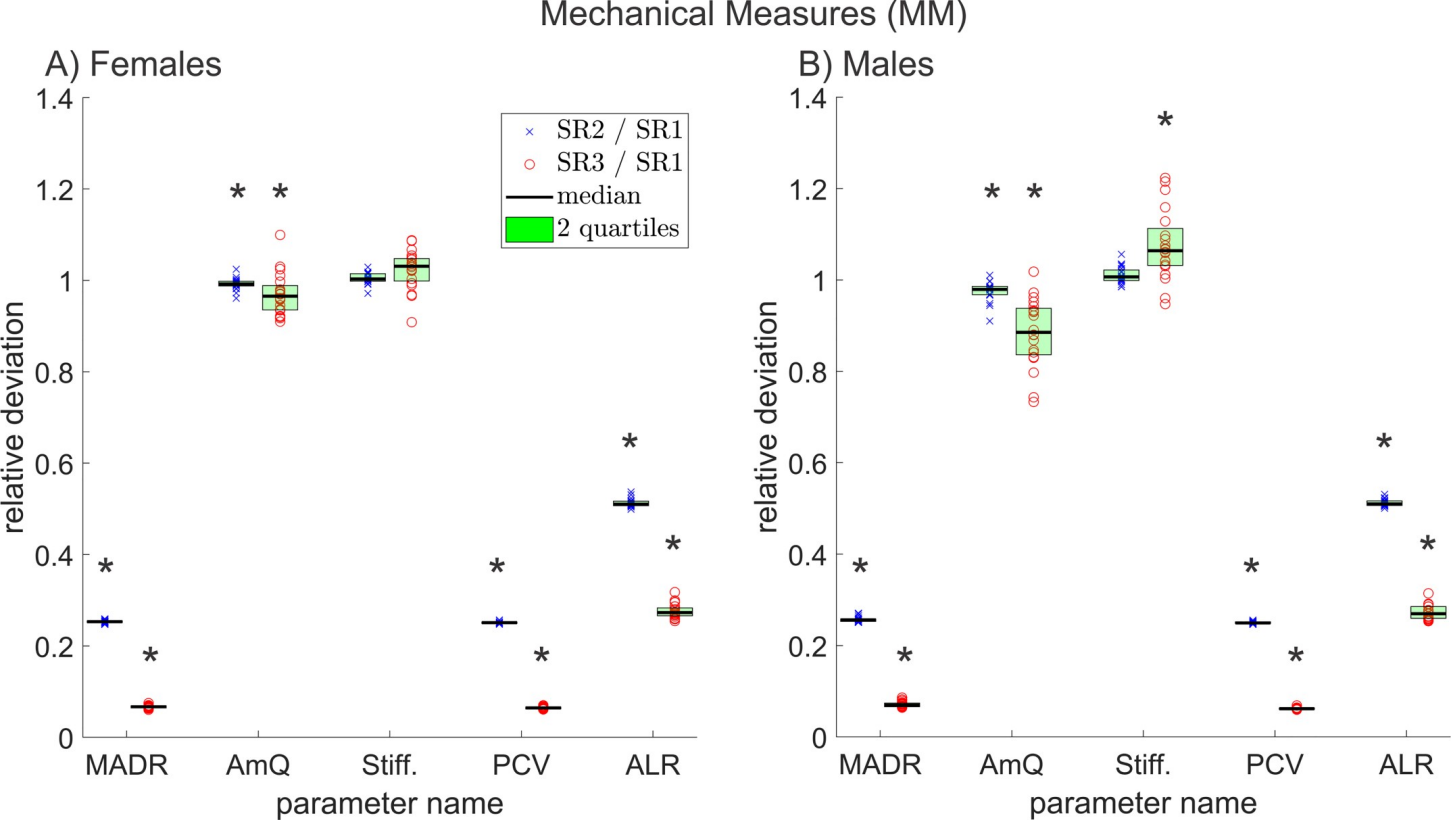
Relative deviations between all mechanical measures, of reduced and original resolution data for A) females and B) males. The * symbols indicate statistically significant deviations (p≤0.05).

Statistical analysis showed clear influences of image resolution on a variety of parameters, although there were distinct differences between the seven different groups of measures. Definitions of parameters vary and in some cases, although the definition is almost identical, the results of the statistical analysis differ. In other cases the definitions look very dissimilar but the results of the statistical analysis are almost the same. However, some of the parameters are related in a simple linear way or their relations are already known. To increase the clarity, we excluded the latter cases from the discussion and only included them in the supporting information. Directly linear or mathematically dependent parameters, which yield identical or almost identical results in the statistical analysis as already discussed parameters, are only briefly mentioned at the end of each subsection. They are also not included in any given numbers of statistically significantly changing parameters. Removed parameters are marked in S1–S3 Tables. Overviews for the relevant parameters of each group are given in Figs 4–10. The structure of these seven figures is as follows:

They illustrate the relative deviation for all parameters in their group separated for A) females and B) males. The relative deviation is calculated by dividing the parameters of the reduced resolutions by the parameters of the original resolution for each data triad. Each x is the relative deviation between one SR2 parameter value and its corresponding SR1 parameter value. Accordingly, each circle is the relative deviation between SR3 and SR1 parameter values. A value of 1 indicates no change; smaller values indicate a decrease in the SR2 or SR3 parameters compared to the SR1 parameter; greater values correspond to an increase. The green area contains the 50% of the 20 data points that showed the least deviation between the different resolutions. An asterisk symbol indicates statistical significant change (p ≤ 0.05). In some cases, parameters could not be depicted in these figures since they reached zero or negative values. In these figures, parameters that reached the value 0 can result in an infinite relative change. The not depicted parameters are *AVI*, *PhAI*, *PhA*, *SpSI*, *SpS* and *GGI*. Furthermore, parameters that were linearly related or directly mathematically dependent from already depicted parameters were not included in Figs 4–10.

The descriptive values of these missing parameters are given in S2 Table, which contains mean, standard deviation, max and min values for all parameters. S3 Table contains the p-values for all Friedman and general linear model repeated measures tests and all performed post-hoc tests for females and males. Parameters that were not included in Figs 4–10 due to their close relations to already depicted parameters were marked in S1, S2 and S3 Tables. All p-values of the general linear model post hoc tests and of the Wilcoxon tests for pairwise comparison were Bonferroni corrected as shown in S2 Fig.

In the following, after a short discussion of computational costs and duration of experiment, all groups of parameters are discussed sequentially. Each subsection begins with the number of the statistically significantly changing relevant parameters (p-value of Friedman test or GLM ≤ 0.05) of this group separated for females and males. Subsequently, conspicuous behavior for all parameters in this group is discussed in the order in which the parameters are shown in the respective figure. Parameters which are not depicted in the figures are discussed last. Furthermore, the observed behavior is explained and a summary of the differences between the parameters calculated for females and males is given. Each subsection is completed by mentioning the not discussed, closely related parameters. In S1 Table all removed parameters are marked. Afterwards, a general summary of all results is given.

### Computational costs and duration of experiment

The computational costs of the segmentation process (without consideration of video loading time) behaves basically as follows: it grows linearly with the number of segmented video frames and number of pixels per video frame and shows a below linear growth for the number of used seed points (*f* = *O*(*n_frames_* ∙ *n_pixel_* ∙ *n_seed points_^τ^*)*τ* < 1) The lower growth for the number of seed points is mainly attributable to the fact that the algorithm already marks the majority of the glottal area for the first seed point and then only has to check for most of the following seed points whether these lie within the marked area. For 1000 frames and peak values of over 1500 seed points used in this study, the segmentation algorithm took about 37 seconds to execute on an intel Core i7-6700 CPU with 32 GB RAM. Due to the required manual adjustments of segmentation settings the segmentation of one video in the original resolution took about 15 to 20 minutes.

### Fundamental period measures (FPM)

*F0* did not show statistically significant deviation for females and males between different resolutions. The relative deviation for *F0* is depicted in Fig 4.

The rather small deviations between *F0* at different resolutions can be explained as follows: While the length of individual cycles may vary between resolutions, lengthening one of the inner cycles always results in a shortening of one of its neighbor cycles, and vice versa. Therefore, the total signal length can only change as much, as the beginning of the first cycle and the ending of the last cycle changes. Respectively, the average cycle length hardly changes and hence *F0*, describing indirectly this average cycle length, does not deviate much between resolutions. As a consequence, it is expected that the changes in *F0* would be even lower if a larger number of consecutive cycles was investigated.

No noticeable differences for the female and male groups were observed.

*MCD* and *F0* are obviously mathematically connected [60] and behaved similarly, see also S1 Table.

### Period perturbation measures (PPM)

Out of 6 relevant PPM only the parameter *PPQ5* deviated statistically significantly between resolutions and only for males between SR3 and SR1. The relative deviations for all relevant PPM are shown in Fig 5.

For *TP*, in contrast to all other calculated PPM, zero perturbation corresponds to the value 1 instead of 0. Since the examined subjects were healthy, the PPM are expected to reach values close to their optimum values. Hence, the relative deviations between the different resolutions for *TP*, which mostly reached values close to one, were much smaller than for the other PPM, which mostly reached values slightly higher than zero.

*Mjit* did not change statistically significantly between resolutions. However, the parameter value still changed distinctly for some subjects.

*PPQ3*, *PPQ5* and *PPQ11* showed relatively strong differences in their reaction on changing resolution in comparison to each other. In particular, it is conspicuous that the deviation does not show a systematic behavior for these three parameters, as one might expect, since the mathematical definitions behind these parameters are based on the same formula [56]. *PPQ* parameters can react very distinctly to different patterns of disturbances in the cycle lengths. If for example every other cycle is slightly prolonged, which is sometimes the case, if the camera sampling rate is not a multiple of the fundamental frequency, *PPQ3* and *PPQ11* will in general be larger than *PPQ5*. (See S1 Proof). This applies also for the *APQ* and *EPQ* measures.

Since *PVI* deviations are calculated quadratic between the cycle lengths, the relative deviations between the resolutions scatter more, for certain cases, in comparison to other parameters. The other important factor one might consider working with *PVI* is that it is independent of the order of values. This means a vector of random values and the same vector sorted from smallest to largest value will yield the exact same *PVI* (see S2 Proof). This also applies to the *AVI*. This means if in some cases the lengths of the single cycles differ between resolutions, both resolutions have the same number of cycles of each length in total, the *PVI* does not change but the other PPM do. Hence, in comparatively many cases the *PVI* did not change at all between different resolutions in contrast to the other PPM.

Even though PPM deviated not statistically significantly in almost all cases, still for some subjects relatively high differences were noted. This is partly due to the fact that in some cases the parameters were only slightly greater than zero. Hence, small absolute differences can lead to large relative differences. Nevertheless, comparatively large absolute deviations were found in a small number of cases (S2 Table also illustrates this since the max values for many Jitter parameters change distinctly between different SRs). This large changes in PPM for some subjects and small changes for others indicate that it depends strongly on the subject, whether a reduction of the resolution causes significant differences in PPM or not.

In males the maximal relative deviations between the PPM for different resolutions seemed to be smaller compared to females, although in general actual deviations in males between PPM calculated for different resolutions seemed to be more frequent. This may be due to the comparatively longer cycles in male GAWs. In longer cycles, a single-frame-jump of maximum positions (that are used for determining cycle start and end) may be more frequent but simultaneously leads to a reduced relative change in length of the cycle.

The measures *Jit(%)*, *JitFac* and *JitRat* are mathematically dependent from *MJit* [60]. *PPF* showed an almost linear dependency of *MJit*. Similarly, *RAPB* and *RAPK* are mathematically identical, except for a cycle number dependent factor [60], and showed a linear dependency from *PPQ3*. Hence all these mathematically dependent measures were not discussed here.

### Amplitude perturbation measures (APM)

All seven relevant APM showed statistically significant deviations for both females and males. The relative deviations are depicted in Fig 6, except for the parameter *AVI* since it can reach negative values and hence, is not suitable for relative comparison as explained previously.

As *TP*, *AP* has an optimum of 1 and therefore smaller relative disparities between the different resolutions than the other APM in Fig 6, all of which have an optimal value of zero.

In contrast to *MJit* and *Jit(%)*, *MShim* and *Shim(%)* differ more, which could be partly due to the problematic definition of *Shim(%)*. As it was pointed out in a previous paper, this definition does not really normalize *MShim* in a useful way [60].

All *APQ* parameters use the same formula as the *PPQ* and *EPQ* parameters; hence, the same restrictions apply for them.

As with the other APM, *AVI* for most subjects also increases with decreasing resolution, indicating an increasing perturbation measured by this parameter.

In total all APM, for females and for males, showed statistical significant deviations between different resolutions (for details see S3 Table). Also, the degree of measured perturbation seemed to increase for the comparison of SR1 and SR3 towards SR1 and SR2. This can be explained by the number of pixels decreasing with the resolution. If the GAW consists of a smaller amount of pixels, relative deviations of the dynamic range increase because the amplitude height can only vary in whole pixels.

In general, the increasing of perturbation measures seemed for males a little more pronounced than for females since for males the deviation between SR1 and SR2 was more often statistically significant than for females. This could be the case because the pixel sizes of the glottides, for the examined males, varied more than for females (see paragraph **Shortcomings**) and in some cases contained less pixels than female glottides. Respectively, the influence of a further decreasing resolution on APM is expected to be stronger for recordings with already very small glottides.

*APF* was excluded since it is for all examined subjects almost exactly 11.5 times *MShim*, although their definitions differ distinctly [53, 56]. S3 Proof explains why both parameters have an almost linear connection for low amplitude perturbation.

### Energy perturbation measures (EPM)

None of the four EPM deviated statistically significantly in females, in males three did. In Fig 7 the relative deviations for the EPM are illustrated analogous to the previous figures.

Since the cycle energy is calculated as sum of squares of all data points within the cycle, energy perturbation is dependent to period and amplitude perturbation. PPM mostly did not change statistically significantly for different resolutions, APM did, and even stronger for males than for females. For that reason EPMs, which are connected to period and amplitude perturbation, changed only statistically significantly for males. In addition, it can be observed that the relative deviations between SR1 and SR3 are again more pronounced than between SR1 and SR2, like it was the case for PPM and APM.

### Symmetry measures (SM)

For females four out of seven relevant SM deviated statistically significantly; for males only two did. In Fig 8, the relative deviations for the parameters *PhAI*, *PhA*, *SpSI* and *SpS* are not shown; again because they can reach zero or negative values. In all other aspects, Fig 8 is similar to the previous figures.

A decrease of a symmetry parameter indicates a decrease in symmetry between the two partial GAWs and each parameter describes a different feature of the partial GAWs as listed in S1 Table. Hence, the decrease of *AmSI* and *AmS* indicates a decrease in symmetry of cycle amplitudes.

*WaSI* changed to a lesser extend between the resolutions, but more consistently for the different subjects. Hence, WaSI still changed statistically significant for males and females.

With the exception of the *SpS* parameter for males, none of the non-plotted SM parameters (*PhAI*, *PhA*, *SpSI* and *SpS*) showed statistically significant changes. Due to apparently small random shifts of cycle maximum positions and changes in overall area between different resolutions, the values between resolutions varied to some extent.

Altogether, the changing SM indicate, that the overall shapes of the partial GAWs change with decreasing resolution. However, the ratio between the areas of the two partial GAWs and the positions of their maxima do not change significantly, nevertheless, for some subjects still more distinct changes of the parameters describing these features were observed, similar to the PPM. The influence on the parameters can be partly attributed to the computation method of the midline. Although the midline was determined for each recording triad using the same specifications, the robustness of the algorithm decreases with decreasing pixel count of the glottal area. This can lead to a changing degree of symmetry between both partial GAWs and hence, to changing symmetry parameters.

For females, more of the parameters changed statistically significantly, which could be attributed to the smaller cycle lengths and hence, a greater influence of period perturbation and a lower robustness of the midline computation.

The definition of *DyRSI* is very similar to the definition of *AmSI* and analogously *DyRS* and *AmS* are similarly defined. To avoid artificially increasing the number of significant parameters *DyRSI* and *DyRS* were excluded.

### Glottal dynamic characteristics (GDC)

Three out of five relevant GDC changed statistically significantly in females and four in males. The parameter *GGI* was unsuitable for a relative comparison, which is why it is not shown in Fig 9. The relative deviations of the remaining relevant GDC are depicted in Fig 9.

*OQ* changed statistically significantly in females and males. The change between resolutions for *CQ* was statistically significant only in males. The decrease of *OQ*, as it can be seen in Fig 9, implies a prolonged glottis closed phase, since *OQ* is defined as Glottis open time/ cycle duration [62,63], see also S1 Table.

The standard deviation of the changes of *PQ* is comparatively large. This is attributed to the usually short plateau length of this quotient, which leads to large relative changes, as this fraction is lengthened or shortened.

With decreasing resolution, the relative proportion of the constantly open glottis area on the maximal area also decreases. This leads to an increase of the *GAI* and a simultaneous decrease of the *GGI* (S2 Table and Fig 9).

The different GDC reacted very dissimilar to the decreasing resolution. This can be attributed to specific changes in the GAW in recordings with lesser resolution towards the original recordings. For example, it appears that in some cases the phase in which the glottis remains closed seems to be prolonged.

*CQ* was the only relevant parameter that changed statistically significantly for males but not for females. This could be explained by the in general longer cycles in male phonation. Since more data points are included in the closing phase of the male phonation cycle, a rather small relative difference between resolutions is more apparent.

*SQ*, *SI*, *RQ* and *AQ* can be calculated directly from *OQ* and *CQ* [60] and hence were excluded.

### Mechanical measures (MM)

All but one of the five relevant mechanical measures changed statistically significant in females; in males, all did. Fig 10 contains the relative deviations of all relevant MM between different resolutions.

*MADR* decreases clearly with decreasing resolution. This is obvious, since it depends on the number of pixels of which the segmented part of the glottis consists of [60] and this number decreases for lower resolutions.

The parameter *AmQ* is a normalized version of *MADR* and is hence, not affected to the same extent by decreasing resolution as *MADR* [60]. Still *AmQ* and *Stiff* change in males statistically significantly; *AmQ* also does in females. A possible explanation could be an increasing steepness of the GAW for lower resolutions since this is the feature *AmQ* and *Stiff* are describing.

*PCV* is, similar to *MADR*, dependent on the number of pixels [60] and behaves therefore, analogous to *MADR*.

The parameter *ALR* is also dependent on the pixel count since it describes the ratio between dynamic range and glottis length (see S1 Table). With decreasing resolution, the dynamic range decreases quadratically, but the glottis length only decreases linearly, which leads to a reduction of the parameter value.

In total, the MM reacted strongly to decreasing resolution, in most cases because the parameters were pixel-dependent. Since pixel dependency is highly problematic for the comparability of those parameters, the use of metric parameters, as replacement, should be considered. Metric parameters of the vocal fold oscillation can e.g. be obtained by laser distance measurement as done by Semmler et al. [17]. The changes between resolutions were more pronounced for the comparison between SR1 and SR3 than SR2 and SR1.

The changes of the GAW slope related parameters (*AmQ* and *Stiff*) were in males even more distinct than in females, which could be attributable to a more pronounced increase in GAW slope in males and would be consistent with also statistically significant change in *OQ* in males. Since the closed phase seems to be prolonged (decrease of *OQ*) and the position of the peak of the cycle doesn’t change significantly in most cases (almost no statistically significant PPM in males) an increased steepness of the GAW seems to be a logical consequence. However, *AmQ* and *Stiff* both only measure the maximum slope between two frames. Hence, also the reduced number of pixels and therefore, the enhanced variations in slope between frames could result in a relatively larger maximum slope.

*PA* was excluded since it depended almost linearly on *PCV* (Pearson correlation coefficient: 0.96).

## Summary

In total, 18 (51%) out of 35 investigated relevant parameters changed in females and 22 (63%) out of 35 in males. From this, we conclude that reducing the resolution of HSV recordings has a substantial impact on the overall shape of the extracted GAW. The least affected groups of measurements were FPM and PPM; although PPM measures showed strong differences in the effect of decreasing resolution for single subjects as seen in Fig 5. The mostly affected groups were APM and MM. Of the investigated SM and GDC, a major part deviated statistically significantly and the biggest differences between females and males were observed in EPM since they changed statistically significantly in males but were mostly unaffected in females. The only group of measurements that (as the results of this study imply) is truly unaffected by decreasing resolution is FPM.

From various changing parameters, it is possible to deduce the following changes in the shape of the GAW at decreasing resolution: The relative perturbation in amplitude increases as all amplitude perturbation measures increase significantly. The pixel count of the GAW obviously decreases with resolution. This is also indicated by significantly changing pixel dependent measures like *MADR*. As it is illustrated in Fig 9, the *Open Quotient* decreases. This implies a shorter open time per cycle and hence a prolonged closed phase (it is hereby assumed that the closed phase is defined as a “true closed phase”, as it was the case in this work, i.e. the glottal area is 0 pixels). Also, the steepness of the GAW slope increases as *AMQ* decreases and *Stiff* increases, both measures of GAW slope.

**Fig 11:**
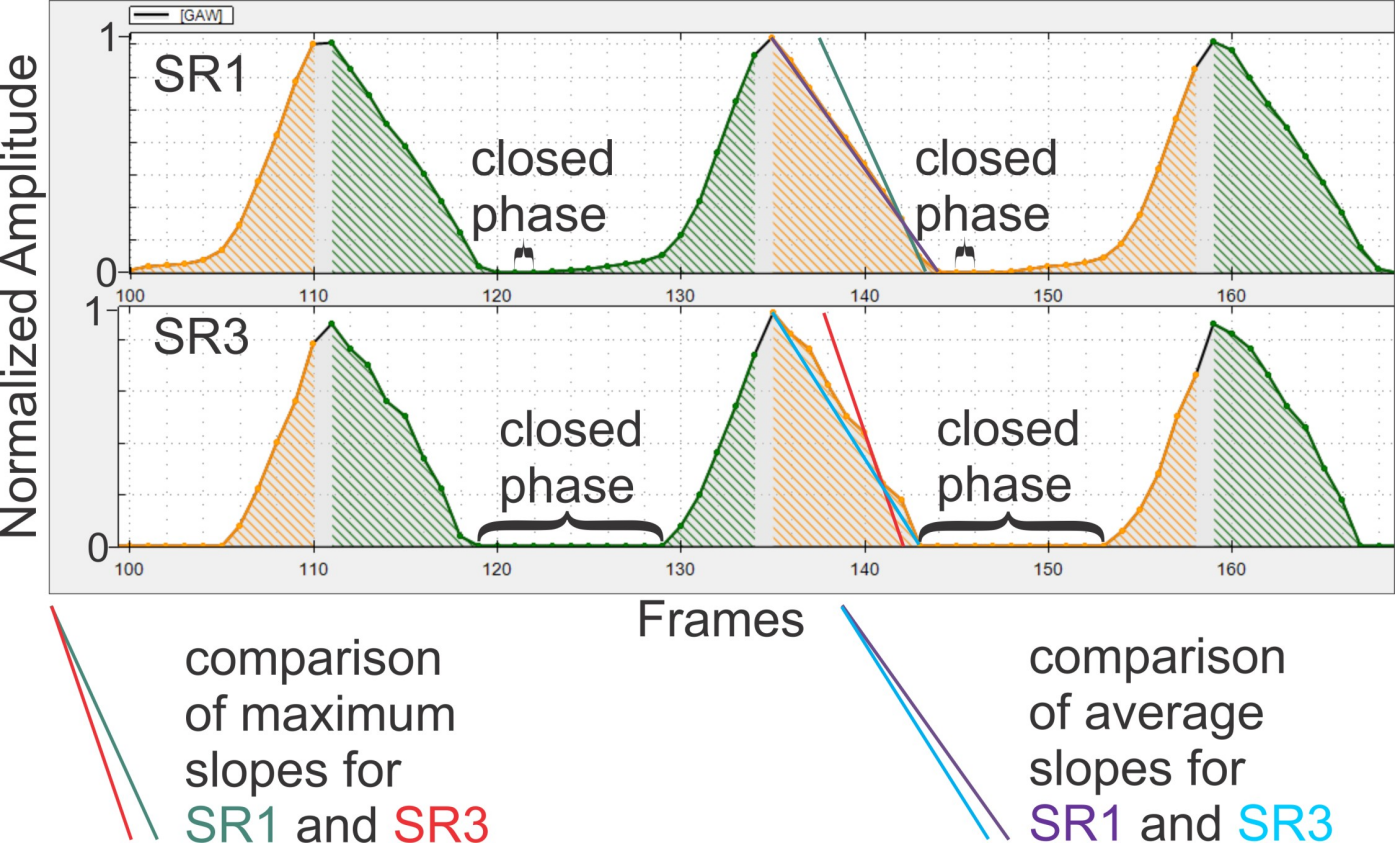
Change in overall shape of a male GAW with decreasing resolution.

In Fig 11 a male GAW (normalized for dynamic range) that showed a comparatively large expansion of its closed phase (and a corresponding decrease in the length of the opening and closing phases) after reducing resolution is depicted for SR1 and SR3. In the recordings with decreased resolution the small open fractions of the glottis during its most closed stage can no longer be detected and hence not segmented as part of the glottal area. This results in the prolonged closed phases in many recordings. In Fig 11, also an increase of maximal and average slope of the closing phase is visible. For better comparability, maximal and average slope of one SR1 and one SR3 cycle in Fig 11 are depicted at the bottom of the figure. This increase in slope is on the one hand a secondary effect of the increased closed phase. On the other hand, a decreased pixel resolution produces a "rougher" GAW, since a single detected or not detected pixel can have a greater influence on the total waveform shape. Hence, the relative maximum slope increases.

Overall, we expect that the lower the spatial resolution, the stronger the changes in parameters, since less and less features of the glottis would be recognizable. Respectively for very high resolutions the influence of changing resolution should decrease (with exception of directly pixel dependent parameters). Since in this study observed influence of changing resolution on the parameters was considerable and care was taken to exclude all other possible influencing factors besides resolution, we expect that this influence would also be observable for other cameras and settings.

In Table 1 the numbers of the relevant statistically significantly changing measures for all seven groups for females and males are summarized. It also contains all relevant examined parameters that could not be depicted in Figs 4–10. Parameters that changed only in females or only in males are highlighted in bold print.

**Table 1:** Summary of the statistical test results for all parameter groups (based on general linear model and Friedman tests).

Name of group	Measures in group	Significantly changing parameters in females	Significantly changing parameters in males	Significantly changing parameters in both
**Fundamental period measures**	1	-	-	-
**Period perturbation measures**	6	-	PPQ5*^1^	-
**Amplitude perturbation measures**	7	AP***, MShim***, Shim(%)***, APQ3***, APQ5***, APQ11***, AVI***	AP***, MShim***, Shim(%)***, APQ3***, APQ5***, APQ11***, AVI***	AP, MShim, Shim(%), APQ3, APQ5, APQ11, AVI
**Energy perturbation measures**	4	-	EPQ3*, EPQ5*, EPF*	-
**Symmetry measures**	7	SpS*, AmSI***, AmS*, WaSI***	AmSI**, WaSI***	WaSI, AmSI
**Glottal dynamic characteristics**	5	OQ***, GGI***, GAI***	OQ***, CQ**, GGI***, GAI***	OQ, GGI, GAI
**Mechanical measures**	5	MADR***, AmQ***, PCV***, ALR***	MADR***, AmQ***, Stiff***, PCV***, ALR***	MADR, AmQ, PCV, ALR
**Total**	35	18	22	16

*: p ≤ 0.05

**: p ≤ 0.01

***: p ≤ 0.001

^1^ Parameters that changed only in females or only in males are highlighted bold print.

## Shortcomings

Several limitations have to be mentioned. The pixel area occupied by the glottis differs between recordings. In our recordings, for men, the GAW maximum fluctuated between 742 and 3214 pixels, for women the range was between 978 and 2057 pixels. This variation can be attributed to different sizes of the glottides but also to a varying distance between endoscope tip and glottis. These effects may be comparable to a changing resolution by pixel averaging. Accordingly, significances may be influenced by "outlier recordings", which had a particularly low or high endoscope-tip glottis distance or glottis size. Also, image noise may be smoothed for lower resolutions, as this is an unavoidable side effect of the applied pixel averaging method. Moreover, the sample size was rather small.

Recordings in this study were only created using a specific camera applying specific settings and only healthy subjects were included. The usage of another recording system could affect the influence of changing resolution. However, the observed influences on parameters in this work were considerable and such variations in camera and settings would not lead to completely different recording results. E.g. changing illumination would affect the observed borders of the glottal area only marginally as long as the recording is not completely dark (or bright). Similarly, a changing frame rate can affect parameters [36], but we expect that the effects would mostly simply add up. However, to make more precise statements, further studies will be required that investigate several simultaneous influences. Hence, we do not expect that for different cameras and different settings the influence of changing spatial resolution will significantly change. Similarly, the exact impact of changing resolution might differ between healthy and disordered subjects and might be dependent on the exact type of disorder. However, investigating all these possible influences was not within scope in this work.

Not all potential parameters were considered. There are more parameters and in some cases they are defined slightly differently under the same name. It should also be noted that in the source material not all parameters are calculated for the GAW but also for related signals like the glottal flow and for the audio signal, although formulas in this work were only applied to the GAW. In particular, it should be mentioned that the different commercial software tools might deviate significantly in the calculation of various parameters [57]. Furthermore, slightly different GAW definitions exist that were not considered [27–30]. Also, the definition of the glottal closed phase slightly differs between works [78–80].

## Conclusion

With this work, another gap in the study of factors influencing GAW parameters was reduced and the understanding of these parameters was improved. It was found that about half of the examined objective GAW parameters were statistically significantly influenced by changing camera spatial resolution. This sensitivity towards the resolution does not only affect the comparability between studies, since the effective resolution also changes with the distance between the glottis and the endoscope tip. That this change probably cannot be neglected is indicated by the number of glottis pixels changing up to fourfold between male recordings. Hence, the comparability of certain parameters between different recordings is not necessarily given when using different spatial camera resolutions.

To counteract these influences by changing resolution, we suggest the following: First, we recommend avoiding the use of the most seriously affected parameters or, if their use is indispensable, compensating for some of the systematic influences by using e.g. a normalized GAW definition. Second, the use of cameras with higher resolution seems reasonable since this allows a comparatively fine resolution of smaller or more distant glottides and hence, a lower disparity of recordings. Third, for look up tables with standard values for parameters, the camera resolution must be specified for which these standard values are valid. Future studies will investigate other potential influencing factors on objective parameters in HSV imaging and finally help to reduce the amount of parameters in use to a smaller and more robust set of parameters. This will improve the comparability of studies and the validity of calculated data.

## Supporting information

S1 Table Parameter information with from statistical analysis excluded parameters highlighted in orange. (DOCX)

S2 Table Descriptive values of all parameters for females and males in all resolutions. (PDF)

S3 Table p-values of all relevant statistical tests performed for females and males. (PDF)

S1 Proof PPQ5 is less than PPQ3 and PPQ11 under certain conditions. (PDF)

S2 Proof PVI and AVI are independent of the order of their elements. (PDF)

S3 Proof Similar behavior of MShim and APF for low perturbation. (PDF)

S1 Fig A) Detection of 20 max based cycles (each cycle starts at a local maximum and ends before the next local maximum) in total and partial GAWs and B) Calculation of in total 50 different cycle based parameters.
(TIF)

S2 Fig Workflow for statistical analysis for each parameter. For each parameter three sets from recordings with different resolutions of 20 values each were calculated. For each set of three sets, the illustrated statistical analysis workflow was performed.
(TIF)
