# Supplementary material for: Influence of spatial camera resolution in high-speed videoendoscopy on laryngeal parameters
Source: PLoS One. 2019 Apr 22;14(4):e0215168. doi: 10.1371/journal.pone.0215168 (PMC6476512; doi:10.1371/journal.pone.0215168)
Supplement: S1 Table — (DOCX) [file pone.0215168.s001.docx]

S1 Table : Parameter information with from statistical analysis excluded parameters highlighted in blue.

| **Parameter (unit) and reference** | **Abbreviation** | **Parameter description** |
| --- | --- | --- |
| 1. **Fundamental period measures (FPM)** | | |
| *Fundamental Frequency* (Hz) | *F0* | Mean value of the reciprocal of the cycle durations |
| *Mean Cycle Duration* (ms) | *MCD* | Mean length of all 20 calculated cycles |
| 1. **Period perturbation measures (PPM)** | | |
| *Time Periodicity* (a.u.)[52] | *TP* | min(T_i_, T_i+1_)/max(T_i_, T_i+1_): describes the deviation in duration between cycle pairs |
| *Mean Jitter* (ms) [53] | *MJit* | Mean deviation in duration between cycle pairs |
| *Jitter (%)*(a.u.) [53] | *Jit(%)* | Normalized mean deviation in duration between cycle pairs |
| *Jitter Factor* (a.u.) [54] | *JitFac* | Normalized mean deviation of reciprocal in duration between cycle pairs |
| *Jitter Ratio* (a.u.) [55] | *JitRat* | Normalized mean deviation in duration between cycle pairs (Jit(%) times 10) |
| *Period Perturbation Quotient-3%* (a.u) [56]^1^ | *PPQ3* | Difference in cycle lengths based on the mean difference between all inner cycles and two neighboring cycles |
| *Period Perturbation Quotient-5%* (a.u.) [56]^1^ | *PPQ5* | Difference in cycle lengths based on the mean difference between all inner cycles and four neighboring cycles |
| *Period Perturbation Quotient-11%* (a.u.) [56]^1^ | *PPQ11* | Difference in cycle lengths based on the mean difference between all inner cycles and ten neighboring cycles |
| *Period Perturbation Factor* (a.u.) [56]^1^ | *PPF* | Mean normalized deviation in duration between cycle pairs |
| *Relative Average Perturbation _Bielamowicz_* (a.u.) [57] | *RAP_B_* | Difference in cycle lengths based on the difference between all inner cycles and two neighboring cycles |
| *Relative Average Perturbation _Koike_* (a.u.) [58] | *RAP_K_* | Normalized difference in cycle lengths based on the difference between all inner cycles and two neighboring cycles |
| *Period Variability Index* (a.u.) [59] | *PVI* | Normalized mean quadratic deviation in duration between each cycle and an average cycle |
| 1. **Amplitude perturbation measures (APM)** | | |
| *Amplitude Periodicity* (a.u.) [52] | *AP* | min(Ai, Ai+1)/max(Ai, Ai+1): describes the deviation in dynamic range between cycle pairs |
| *Mean Shimmer* (dB) [53] | *MShim* | Mean logarithmic deviation in dynamic range between cycle pairs |
| *Shimmer (%)* (dB/log10(pixel)) [60] | *Shim(%)* | Normalized mean logarithmic deviation in dynamic range between cycle pairs |
| *Amplitude Perturbation Quotient-3%* (a.u.) [56]^1^ | *APQ3* | Difference in dynamic range based on the mean difference between all inner cycles and two neighboring cycles |
| *Amplitude Perturbation Quotient-5%* (a.u.) [56]^1^ | *APQ5* | Difference in dynamic range based on the mean difference between all inner cycles and four neighboring cycles |
| *Amplitude Perturbation Quotient-11%* (a.u.) [56]^1^ | *APQ11* | Difference in dynamic range based on the mean difference between all inner cycles and ten neighboring cycles |
| *Amplitude Perturbation Factor* (a.u.) [56]^1^ | *APF* | Mean normalized deviation in dynamic range between cycle pairs |
| *Amplitude Variability Index* (dB) [59] | *AVI* | Logarithmic normalized mean quadratic deviation in dynamic range between each cycle and an average cycle |
| 1. **Energy perturbation measures (EPM)** | | |
| *Energy Perturbation Quotient-3%* (a.u.) [56]^1^ | *EPQ3* | Difference in energy based on the mean difference between all inner cycles and two neighboring cycles |
| *Energy Perturbation Quotient-5%* (a.u.) [56]^1^ | *EPQ5* | Difference in energy based on the mean difference between all inner cycles and four neighboring cycles |
| *Energy Perturbation Quotient-11%* (a.u.) [56]^1^ | *EPQ11* | Difference in energy based on the mean difference between all inner cycles and ten neighboring cycles |
| *Energy Perturbation Factor* (a.u.) [56]^1^ | *EPF* | Mean normalized deviation in energy between cycle pairs |
| 1. **Symmetry measures (SM)** | | |
| *Phase Asymmetry Index* (a.u.) [61]^2^ | *PhAI* | Difference in phase of GAW_L_ and GAW_R_ (side independent) \|position of minimum of GAW_L_ - position of minimum of GAW_R_\| / cycle length (side independent) ^3^ |
| *Phase Asymmetry* (a.u.) [61]^2^ | *PhA* | Difference in phase of GAW_L_ and GAW_R_  (position of minimum of GAW_L_ - position of minimum of GAW_R_) / cycle length |
| *Spatial Symmetry Index* (a.u.) [61] | *SpSI* | Difference in area of GAW_L_ and GAW_R_  (side independent)^3^ |
| *Spatial Symmetry* (a.u.) [61] | *SpS* | Difference in area of GAW_L_ and GAW_R_ |
| *Amplitude Symmetry Index* (a.u.)^4^ | *AmSI* | Difference in maximal glottal area of GAW_L_ and GAW_R_ (side independent)^3^ |
| *Amplitude Symmetry* (a.u.) [61] | *AmS* | Difference in maximal glottal area of GAW_L_ and GAW_R_ |
| *Dynamic Range Symmetry Index* (a.u.) [61] | *DyRSI* | Difference in dynamic range of GAW_L_ and GAW_R_ (side independent) ^3^ |
| *Dynamic Range Symmetry* (a.u.) [61] | *DyRS* | Difference in dynamic range of GAW_L_ and GAW_R_ |
| *Waveform Symmetry Index* (a.u.) [61] | *WaSI* | Overall difference in shape of GAW_L_ and GAW_R_ (side independent)3 |
| 1. **Glottal dynamic characteristics (GDC)** | | |
| *Open Quotient* (a.u.) [62, 63] | *OQ* | Glottis open time/ cycle duration |
| *Closing Quotient* (a.u.) [64] | *CQ* | Glottis closing time/ cycle duration |
| *Speed Quotient* (a.u.) [62, 63] | *SQ* | Glottis opening time/ glottis closing time |
| *Speed Index* (a.u.) [62] | *SI* | (Speed Quotient -1)/ (Speed Quotient +1) |
| *Rate Quotient* (a.u.) [62] | *RQ* | (Glottis closed time + glottis opening time)/ glottis closing time |
| *Asymmetry Quotient* (a.u.) [65]^5^ | *AQ* | (Speed Quotient)/ (1+ Speed Quotient) |
| *Glottis Gap Index* (a.u.) [66]^5^ | *GGI* | Minimum glottal area/maximum glottal area |
| *Plateau Quotient* (a.u.) [67] | *PQ* | Duration during which the glottal area has more than 95% of its maximum/cycle duration |
| *Glottal Area Index* (a.u.) [68] | *GAI* | Dynamic range/(maximum of glottal area times Open Quotient) |
| 1. **Mechanical measures (MM)** | | |
| *Maximum Area Declination Rate* (Mpx/s) [60]^4^ | *MADR* | Maximum closing velocity of one GAW cycle |
| *Amplitude Quotient* (ms) [60] | *AmQ* | (Maximum Area Declination Rate / dynamic range)×1000 |
| *Stiffness* (1/s) [69] | *Stiff* | ratio of maximum instantaneous velocity to movement amplitude in one cycle |
| *Peak Closing Velocity* (Mpx/s) [60] | *PCV* | Estimates the maximum glottis opening or closing speed of one cycle by approximating the GAW as sin wave |
| *Peak Acceleration* (Mpx/(s)^2^) [60] | *PA* | Estimates the maximum glottis opening or closing acceleration for one cycle by approximating the GAW as sin wave |
| *Amplitude-to-Length Ratio* (a.u.) [70] | *ALR* | Dynamic range of glottis/ length of glottis (dynamic range and length both measured in pixels) |

^1^ in the source material one formula is given as “Perturbation Quotient” and one as “Perturbation Factor”. The different types of Perturbation Quotients and Factors in this work were calculated by inserting cycle lengths, dynamic ranges and cycle energies in these original formulas for, in case of the Perturbation Quotient, values of k of 3, 5 and 11. ^2^ The minimum instead of the maximum position of the cycle was used, since the cycle detection was max based. ^3^ Side dependent versions of symmetry measures give information about the direction of an asymmetric behavior. However, since they are calculated cycle-wise and then averaged over all cycles, left and right-sided asymmetries can cancel each other out. For this reason, side independent versions of the measures exist. They can give information about the actual size of an asymmetric effect, but not about the direction (See also [61]).  ^4^AmSI = (min(max[left glottal area], max[right glottal area]))/ (max(max[left glottal area], max[right glottal area]))) ^5^ In the source material the “Glottis Gap Index” is named “Glottis Closure Index”. Similarly the “Asymmetry Quotient” is named “Asymmetry Coefficient”.
