# Supplementary material for: Influence of spatial camera resolution in high-speed videoendoscopy on laryngeal parameters
Source: PLoS One. 2019 Apr 22;14(4):e0215168. doi: 10.1371/journal.pone.0215168 (PMC6476512; doi:10.1371/journal.pone.0215168)
Supplement: S2 Table — (PDF) [file pone.0215168.s002.pdf]

S1 Table

Table S1:. Descriptive values of all parameters for females and males in all resolutions (mathematically dependent parameters are marked in blue) part 1

Parameter name and resolution	Average females/males	Standard deviation females/males	Maximum females/males	Minimum females/males
**Fundamental Period Measures**				
$F0_{SR1}$	311.148 / 170.864	65.566 / 48.941	428.889 / 311.722	232.026 / 107.008
$F0_{SR2}$	311.315 / 170.882	65.558 / 48.820	428.889 / 310.440	232.680 / 107.023
$F0_{SR3}$	311.167 / 170.883	65.382 / 48.935	428.889 / 310.440	232.026 / 106.985
MCD_{SR1}	3.345 / 6.258	0.646 / 1.563	4.313 / 9.350	2.338 / 3.213
MCD_{SR2}	3.343 / 6.257	0.645 / 1.562	4.300 / 9.350	2.338 / 3.225
MCD_{SR3}	3.344 / 6.260	0.643 / 1.565	4.313 / 9.350	2.338 / 3.225
**Period Perturbation Measures**				
TP_{SR1}	0.963 / 0.974	0.021 / 0.011	0.996 / 0.994	0.921 / 0.948
TP_{SR2}	0.962 / 0.973	0.022 / 0.011	0.992 / 0.994	0.911 / 0.955
TP_{SR3}	0.960 / 0.968	0.022 / 0.017	0.993 / 0.995	0.905 / 0.929
$MJit_{SR1}$	0.126 / 0.166	0.065 / 0.078	0.224 / 0.329	0.013 / 0.026
$MJit_{SR2}$	0.126 / 0.174	0.065 / 0.090	0.224 / 0.382	0.026 / 0.026
$MJit_{SR3}$	0.133 / 0.203	0.061 / 0.117	0.237 / 0.447	0.026 / 0.026
$Jit(\%)_{SR1}$	3.919 / 2.668	2.263 / 1.122	8.354 / 5.356	0.352 / 0.555
$Jit(\%)_{SR2}$	3.955 / 2.738	2.348 / 1.164	9.468 / 4.620	0.807 / 0.555
$Jit(\%)_{SR3}$	4.215 / 3.290	2.363 / 1.834	9.972 / 7.469	0.699 / 0.528
$JitFac_{SR1}$	3.882 / 2.672	2.232 / 1.124	8.267 / 5.367	0.375 / 0.583
$JitFac_{SR2}$	3.909 / 2.742	2.324 / 1.162	9.369 / 4.644	0.755 / 0.583
$JitFac_{SR3}$	4.183 / 3.298	2.357 / 1.840	9.972 / 7.505	0.660 / 0.553
$JitRat_{SR1}$	39.189 / 26.680	22.632 / 11.216	83.542 / 53.563	3.521 / 5.555
$JitRat_{SR2}$	39.554 / 27.381	23.479 / 11.644	94.681 / 46.197	8.066 / 5.555
$JitRat_{SR3}$	42.149 / 32.904	23.625 / 18.339	99.723 / 74.688	6.994 / 5.276
$PPQ3_{SR1}$	2.786 / 1.802	1.626 / 0.776	6.006 / 3.909	0.134 / 0.420
$PPQ3_{SR2}$	2.795 / 1.895	1.663 / 0.809	6.636 / 3.299	0.588 / 0.420
$PPQ3_{SR3}$	2.991 / 2.221	1.675 / 1.231	7.027 / 5.303	0.512 / 0.399
$PPQ5_{SR1}$	2.839 / 1.697	1.405 / 0.599	5.405 / 2.616	0.090 / 0.567
$PPQ5_{SR2}$	2.867 / 1.803	1.318 / 0.686	5.405 / 2.980	0.808 / 0.567
$PPQ5_{SR3}$	2.890 / 2.137	1.292 / 1.067	5.405 / 4.210	0.526 / 0.539
$PPQ11_{SR1}$	2.883 / 1.829	1.568 / 0.707	6.078 / 3.763	0.068 / 0.863
$PPQ11_{SR2}$	3.031 / 2.071	1.337 / 0.808	5.977 / 4.025	1.136 / 0.855
$PPQ11_{SR3}$	2.961 / 2.262	1.520 / 1.112	5.769 / 4.313	0.136 / 0.321
PPF_{SR1}	3.902 / 2.670	2.249 / 1.125	8.304 / 5.394	0.351 / 0.569
PPF_{SR2}	3.937 / 2.744	2.337 / 1.165	9.415 / 4.640	0.781 / 0.569
PPF_{SR3}	4.207 / 3.301	2.363 / 1.844	10.000 / 7.521	0.680 / 0.540
RAP_{BSR1}	0.024 / 0.015	0.014 / 0.007	0.051 / 0.033	0.001 / 0.004
RAP_{BSR2}	0.024 / 0.016	0.014 / 0.007	0.056 / 0.028	0.005 / 0.004
RAP_{BSR3}	0.025 / 0.019	0.014 / 0.010	0.060 / 0.045	0.004 / 0.003
RAP_{KSR1}	0.026 / 0.017	0.015 / 0.007	0.057 / 0.037	0.001 / 0.004
RAP_{KSR2}	0.026 / 0.018	0.016 / 0.008	0.063 / 0.031	0.006 / 0.004
RAP_{KSR3}	0.028 / 0.021	0.016 / 0.012	0.066 / 0.050	0.005 / 0.004
PVI_{SR1}	1.108 / 0.522	0.655 / 0.323	2.771 / 1.378	0.213 / 0.132
PVI_{SR2}	1.118 / 0.553	0.640 / 0.337	2.771 / 1.366	0.279 / 0.132
PVI_{SR3}	1.250 / 0.792	0.802 / 0.667	2.959 / 2.650	0.210 / 0.119

Table S1. Descriptive values of all parameters for females and males in all resolutions (mathematically dependent parameters are marked in blue) part 2

Parameter name and resolution	Average females/males	Standard deviation females/males	Maximum females/males	Minimum females/males
**Amplitude Perturbation Measures**				
AP_{SR1}	0.985 / 0.988	0.005 / 0.003	0.993 / 0.993	0.971 / 0.982
AP_{SR2}	0.984 / 0.986	0.005 / 0.004	0.992 / 0.993	0.974 / 0.980
AP_{SR3}	0.978 / 0.981	0.007 / 0.006	0.990 / 0.990	0.965 / 0.970
$MShim_{SR1}$	0.130 / 0.102	0.049 / 0.030	0.259 / 0.154	0.063 / 0.059
$MShim_{SR2}$	0.140 / 0.122	0.046 / 0.038	0.229 / 0.180	0.072 / 0.062
$MShim_{SR3}$	0.193 / 0.167	0.063 / 0.054	0.314 / 0.264	0.087 / 0.088
$Shim(\%)_{SR1}$	0.211 / 0.164	0.081 / 0.053	0.439 / 0.263	0.098 / 0.090
$Shim(\%)_{SR2}$	0.283 / 0.243	0.095 / 0.080	0.457 / 0.374	0.137 / 0.116
$Shim(\%)_{SR3}$	0.515 / 0.439	0.187 / 0.157	0.944 / 0.750	0.220 / 0.215
$APQ3_{SR1}$	0.906 / 0.665	0.437 / 0.213	2.082 / 0.994	0.260 / 0.351
$APQ3_{SR2}$	0.959 / 0.801	0.404 / 0.265	1.797 / 1.194	0.421 / 0.362
$APQ3_{SR3}$	1.339 / 1.072	0.499 / 0.330	2.248 / 1.474	0.455 / 0.535
$APQ5_{SR1}$	0.949 / 0.778	0.342 / 0.218	1.575 / 1.332	0.425 / 0.412
$APQ5_{SR2}$	1.048 / 0.949	0.354 / 0.288	1.701 / 1.458	0.522 / 0.531
$APQ5_{SR3}$	1.593 / 1.337	0.568 / 0.455	2.815 / 2.107	0.771 / 0.633
$APQ11_{SR1}$	1.177 / 1.050	0.324 / 0.370	1.833 / 2.076	0.723 / 0.472
$APQ11_{SR2}$	1.390 / 1.248	0.465 / 0.446	2.207 / 2.371	0.589 / 0.685
$APQ11_{SR3}$	1.970 / 1.958	0.977 / 0.790	4.272 / 3.695	0.829 / 0.612
APF_{SR1}	1.496 / 1.175	0.559 / 0.350	2.980 / 1.778	0.719 / 0.678
APF_{SR2}	1.615 / 1.402	0.534 / 0.433	2.635 / 2.065	0.831 / 0.709
APF_{SR3}	2.225 / 1.923	0.723 / 0.625	3.621 / 3.066	0.996 / 1.016
AVI_{SR1}	-0.562 / -0.522	0.255 / 0.399	-0.116 / 0.062	-1.092 / -1.434
AVI_{SR2}	-0.452 / -0.441	0.317 / 0.413	0.096 / 0.148	-1.136 / -1.295
AVI_{SR3}	-0.165 / -0.139	0.394 / 0.408	0.590 / 0.525	-0.779 / -1.059
**Energy Perturbation Measures**				
$EPQ3_{SR1}$	7.738 / 6.057	4.259 / 1.910	13.614 / 10.382	1.683 / 1.673
$EPQ3_{SR2}$	7.832 / 6.663	4.354 / 2.393	15.391 / 10.343	1.901 / 1.755
$EPQ3_{SR3}$	8.508 / 7.695	4.153 / 3.086	14.319 / 12.490	2.084 / 2.262
$EPQ5_{SR1}$	7.857 / 5.913	3.590 / 1.944	12.364 / 8.660	1.387 / 2.115
$EPQ5_{SR2}$	8.018 / 6.415	3.500 / 2.176	12.696 / 10.545	2.468 / 2.223
$EPQ5_{SR3}$	8.224 / 7.408	3.443 / 2.671	14.240 / 11.955	2.059 / 2.526
$EPQ11_{SR1}$	8.046 / 6.327	3.912 / 2.337	13.703 / 11.349	1.379 / 2.432
$EPQ11_{SR2}$	8.374 / 7.222	3.454 / 2.495	14.612 / 12.028	2.499 / 2.586
$EPQ11_{SR3}$	8.750 / 7.634	4.019 / 2.912	14.581 / 11.641	1.700 / 1.217
EPF_{SR1}	11.296 / 9.460	6.026 / 2.836	19.702 / 15.004	2.879 / 2.586
EPF_{SR2}	11.541 / 10.184	6.219 / 3.598	22.005 / 15.384	2.911 / 2.818
EPF_{SR3}	12.545 / 12.019	5.937 / 4.753	20.670 / 18.763	3.037 / 3.603

Table S1. Descriptive values of all parameters for females and males in all resolutions (mathematically dependent parameters are marked in blue) part 3

Parameter name and resolution	Average females/males	Standard deviation females/males	Maximum females/males	Minimum females/males
**Symmetry Measures**				
$PhAI_{SR1}$	0.027 / 0.039	0.023 / 0.038	0.087 / 0.148	0.000 / 0.000
$PhAI_{SR2}$	0.038 / 0.033	0.022 / 0.044	0.068 / 0.174	0.000 / 0.000
$PhAI_{SR3}$	0.044 / 0.040	0.036 / 0.072	0.100 / 0.299	0.000 / 0.000
PhA_{SR1}	-0.012 / -0.004	0.033 / 0.048	0.049 / 0.066	-0.087 / -0.148
PhA_{SR2}	-0.017 / 0.003	0.030 / 0.037	0.032 / 0.086	-0.068 / -0.105
PhA_{SR3}	-0.008 / 0.004	0.025 / 0.032	0.043 / 0.090	-0.068 / -0.070
$SpSI_{SR1}$	0.056 / 0.054	0.042 / 0.033	0.144 / 0.142	0.010 / 0.015
$SpSI_{SR2}$	0.054 / 0.070	0.037 / 0.054	0.140 / 0.210	0.019 / 0.025
$SpSI_{SR3}$	0.062 / 0.069	0.031 / 0.049	0.174 / 0.236	0.019 / 0.019
SpS_{SR1}	0.009 / -0.012	0.070 / 0.061	0.144 / 0.088	-0.134 / -0.142
SpS_{SR2}	-0.018 / 0.011	0.059 / 0.088	0.112 / 0.210	-0.140 / -0.104
SpS_{SR3}	-0.024 / -0.010	0.059 / 0.078	0.075 / 0.119	-0.174 / -0.236
$AmSI_{SR1}$	0.920 / 0.928	0.056 / 0.048	0.975 / 0.977	0.819 / 0.804
$AmSI_{SR2}$	0.920 / 0.903	0.050 / 0.086	0.978 / 0.971	0.793 / 0.632
$AmSI_{SR3}$	0.880 / 0.855	0.043 / 0.117	0.936 / 0.957	0.765 / 0.457
AmS_{SR1}	1.032 / 0.990	0.105 / 0.092	1.208 / 1.195	0.819 / 0.804
AmS_{SR2}	0.983 / 1.037	0.084 / 0.178	1.137 / 1.600	0.793 / 0.859
AmS_{SR3}	0.952 / 1.000	0.094 / 0.182	1.121 / 1.453	0.765 / 0.457
$DyRSI_{SR1}$	0.903 / 0.920	0.062 / 0.062	0.974 / 0.976	0.742 / 0.754
$DyRSI_{SR2}$	0.905 / 0.901	0.053 / 0.095	0.967 / 0.971	0.785 / 0.605
$DyRSI_{SR3}$	0.883 / 0.855	0.047 / 0.117	0.952 / 0.957	0.765 / 0.457
$DyRS_{SR1}$	1.056 / 0.993	0.128 / 0.109	1.352 / 1.259	0.854 / 0.754
$DyRS_{SR2}$	1.009 / 1.031	0.113 / 0.197	1.280 / 1.672	0.796 / 0.798
$DyRS_{SR3}$	0.953 / 1.002	0.090 / 0.183	1.121 / 1.453	0.765 / 0.457
$WaSI_{SR1}$	0.971 / 0.951	0.026 / 0.058	0.999 / 0.997	0.911 / 0.817
$WaSI_{SR2}$	0.968 / 0.951	0.028 / 0.054	0.997 / 0.998	0.899 / 0.833
$WaSI_{SR3}$	0.961 / 0.944	0.029 / 0.066	0.996 / 0.992	0.901 / 0.732

Table S1. Descriptive values of all parameters for females and males in all resolutions (mathematically dependent parameters are marked in blue) part 4

Parameter name and resolution	Average females/males	Standard deviation females/males	Maximum females/males	Minimum females/males
**Glottal dynamic characteristics**				
OQ_{SR1}	0.999 / 0.927	0.001 / 0.134	1.000 / 1.000	0.995 / 0.559
OQ_{SR2}	0.992 / 0.887	0.018 / 0.139	1.000 / 1.000	0.915 / 0.542
OQ_{SR3}	0.971 / 0.799	0.045 / 0.182	1.000 / 0.996	0.785 / 0.500
CQ_{SR1}	0.489 / 0.412	0.066 / 0.083	0.598 / 0.556	0.333 / 0.275
CQ_{SR2}	0.493 / 0.401	0.070 / 0.084	0.605 / 0.530	0.302 / 0.275
CQ_{SR3}	0.488 / 0.373	0.070 / 0.093	0.613 / 0.523	0.313 / 0.248
SQ_{SR1}	1.090 / 1.303	0.315 / 0.390	2.017 / 2.292	0.678 / 0.772
SQ_{SR2}	1.060 / 1.259	0.317 / 0.328	2.037 / 2.061	0.659 / 0.790
SQ_{SR3}	1.034 / 1.179	0.241 / 0.266	1.525 / 1.816	0.626 / 0.707
SI_{SR1}	0.022 / 0.107	0.131 / 0.135	0.333 / 0.391	-0.196 / -0.130
SI_{SR2}	0.007 / 0.094	0.133 / 0.123	0.340 / 0.344	-0.210 / -0.119
SI_{SR3}	-0.003 / 0.064	0.119 / 0.112	0.202 / 0.287	-0.236 / -0.173
RQ_{SR1}	1.092 / 1.538	0.314 / 0.544	2.017 / 2.650	0.678 / 0.802
RQ_{SR2}	1.083 / 1.618	0.365 / 0.571	2.320 / 2.650	0.659 / 0.892
RQ_{SR3}	1.109 / 1.873	0.347 / 0.752	2.217 / 3.053	0.639 / 0.919
AQ_{SR1}	0.511 / 0.554	0.066 / 0.068	0.667 / 0.696	0.402 / 0.435
AQ_{SR2}	0.503 / 0.547	0.066 / 0.062	0.670 / 0.672	0.395 / 0.440
AQ_{SR3}	0.498 / 0.532	0.059 / 0.056	0.601 / 0.644	0.382 / 0.413
GGI_{SR1}	0.139 / 0.049	0.083 / 0.081	0.276 / 0.318	0.002 / 0.000
GGI_{SR2}	0.135 / 0.046	0.081 / 0.077	0.263 / 0.294	0.000 / 0.000
GGI_{SR3}	0.115 / 0.039	0.074 / 0.064	0.237 / 0.219	0.000 / 0.000
PQ_{SR1}	0.128 / 0.104	0.025 / 0.025	0.173 / 0.141	0.088 / 0.069
PQ_{SR2}	0.127 / 0.106	0.021 / 0.025	0.172 / 0.146	0.095 / 0.066
PQ_{SR3}	0.120 / 0.118	0.019 / 0.032	0.157 / 0.179	0.086 / 0.059
GAI_{SR1}	0.861 / 1.061	0.083 / 0.260	0.998 / 1.793	0.724 / 0.682
GAI_{SR2}	0.873 / 1.117	0.091 / 0.275	1.094 / 1.847	0.740 / 0.706
GAI_{SR3}	0.916 / 1.283	0.113 / 0.370	1.276 / 2.007	0.769 / 0.784
**Mechanical Measures**				
$MADR_{SR1}$	1.294 / 0.961	0.301 / 0.376	1.796 / 1.791	0.538 / 0.394
$MADR_{SR2}$	0.327 / 0.246	0.075 / 0.093	0.455 / 0.448	0.137 / 0.107
$MADR_{SR3}$	0.086 / 0.067	0.019 / 0.024	0.117 / 0.116	0.037 / 0.032
AmQ_{SR1}	1.000 / 1.540	0.151 / 0.318	1.374 / 2.395	0.823 / 1.009
AmQ_{SR2}	0.992 / 0.001	0.148 / 0.000	1.320 / 0.002	0.822 / 0.001
AmQ_{SR3}	0.967 / 0.001	0.131 / 0.000	1.260 / 0.002	0.831 / 0.001
$Stiff_{SR1}$	1160.614 / 811.380	190.332 / 164.237	1539.750 / 1128.767	781.158 / 559.879
$Stiff_{SR2}$	1165.049 / 818.579	187.022 / 161.628	1543.236 / 1137.156	778.992 / 558.952
$Stiff_{SR3}$	1183.715 / 866.749	175.426 / 156.029	1523.412 / 1195.495	833.326 / 614.218
PCV_{SR1}	1.249 / 0.748	0.381 / 0.270	2.048 / 1.253	0.510 / 0.320
PCV_{SR2}	0.314 / 0.187	0.096 / 0.067	0.515 / 0.313	0.131 / 0.079
PCV_{SR3}	0.080 / 0.046	0.024 / 0.017	0.130 / 0.079	0.034 / 0.020
PA_{SR1}	2539.654 / 828.449	1193.097 / 413.806	5533.619 / 1591.518	754.365 / 243.401
PA_{SR2}	638.392 / 207.544	302.053 / 104.440	1391.680 /	193.374 / 60.530
PA_{SR3}	164.127 / 51.727	77.758 / 26.765	350.824 / 109.148	51.084 / 15.094
ALR_{SR1}	11.754 / 12.538	2.214 / 3.710	15.932 / 20.796	8.574 / 6.497
ALR_{SR2}	6.024 / 6.412	1.125 / 1.854	8.088 / 10.406	4.480 / 3.362
ALR_{SR3}	3.232 / 3.395	0.545 / 0.898	4.055 / 5.267	2.456 / 1.888
