# Supplementary material for: Influence of spatial camera resolution in high-speed videoendoscopy on laryngeal parameters
Source: PLoS One. 2019 Apr 22;14(4):e0215168. doi: 10.1371/journal.pone.0215168 (PMC6476512; doi:10.1371/journal.pone.0215168)
Supplement: S3 Table — (PDF) [file pone.0215168.s003.pdf]

S2 Table

Table S2. p-values of all relevant statistical tests performed for females part 1.

Parameter name	p-Value comparison RS1 and RS2	p-Value comparison RS1 and RS3	p-Value comparison RS2 and RS3	p-Value Friedman or General linear model
**Fundamental Period Measures**				
$F0$	-	-	-	0.358
MCD	-		-	0.515^{++}
**Period Perturbation Measures**				
TP	-	-	-	0.352^{++}
$MJit$	-	-	-	0.529
$Jit(\%)$	-	-	-	0.349^{++}
$JitFac$	-	-	-	0.327^{++}
$JitRat$	-	-	-	0.349^{++}
$PPQ3$	-	-	-	0.318^{++}
$PPQ5$	-	-	-	0.858^{++}
$PPQ11$	-	-	-	0.495^{+}
PPF	-	-	-	0.329^{++}
RAP_B	-	-	-	0.320^{++}
RAP_K	-	-	-	0.320^{++}
PVI	-	-	-	0.823
**Amplitude Perturbation Measures**				
AP	0.641^{+}	0.000^{+}	0.003^{+}	0.000^{++}
$MShim$	0.641^{+}	0.000^{+}	0.003^{+}	0.000^{++}
$Shim(\%)$	0.000^{+}	0.000^{+}	0.000^{+}	0.000^{++}
$APQ3$	1.000^{+}	0.000^{+}	0.008^{+}	0.000^{++}
$APQ5$	0.488^{+}	0.000^{+}	0.001^{+}	0.000^{++}
$APQ11$	0.046	0.003	0.033	0.000
APF	0.646^{+}	0.000^{+}	0.003^{+}	0.000^{++}
AVI	0.032^{+}	0.000^{+}	0.005^{+}	0.000^{+}
**Energy Perturbation Measures**				
$EPQ3$	-	-	-	0.086
$EPQ5$	-	-	-	0.461^{++}
$EPQ11$	-	-	-	0.185^{+}
EPF	-	-	-	0.116
**Symmetry Measures**				
$PhAI$	-	-	-	0.092
PhA	-	-	-	0.368^{++}
$SpSI$	-	-	-	0.116
SpS	0.006^{+}	0.107^{+}	1.000^{+}	0.032^{++}
$AmSI$	1.000	0.005	0.005	0.000
AmS	0.004^{+}	0.024^{+}	0.733^{+}	0.012^{++}
$DyRSI$	1.000	0.083	0.219	0.008
$DyRS$	0.004^{+}	0.011^{+}	0.215^{+}	0.006^{++}
$WaSI$	0.008	0.005	0.041	0.000

+ Data was assumed to be normally distributed ++ Greenhouse-Geisser correction was applied. Green: p≤0.05, Red: p>0.05, Blue: parameter was mathematically dependent.

Table S2. p-values of all relevant statistical tests performed for females part 2.

Parameter name	p-Value comparison RS1 and RS2	p-Value comparison RS1 and RS3	p-Value comparison RS2 and RS3	p-Value Friedman or General linear model
**Glottal dynamic characteristics**				
OQ	0.010	0.001	0.001	0.000
CQ	-	-	-	0.229^{+}
SQ	0.005	0.131	1.000	0.040
SI	0.001^{+}	0.040^{+}	1.000^{+}	0.033^{++}
RQ	-	-	-	0.054
AQ	0.001^{+}	0.040^{+}	1.000^{+}	0.033^{++}
GGI	0.007^{+}	0.000^{+}	0.000^{+}	0.000^{++}
PQ	-	-	-	0.069^{++}
GAI	0.000	0.000	0.000	0.000
**Mechanical Measures**				
$MADR$	0.000^{+}	0.000^{+}	0.000^{+}	0.000^{++}
AmQ	0.019	0.015	0.068	0.001
$Stiff$	-	-	-	0.054^{++}
PCV	0.000^{+}	0.000^{+}	0.000^{+}	0.000^{++}
PA	0.000^{+}	0.000^{+}	0.000^{+}	0.000^{++}
ALR	0.000^{+}	0.000^{+}	0.000^{+}	0.000^{++}

+ Data was assumed to be normally distributed ++ Greenhouse-Geisser correction was applied. Green: $p \leq 0.05$, Red: $p > 0.05$, Blue: parameter was mathematically dependent.

Table S2. p-values of all relevant statistical tests performed for males part 1.

Parameter name	p-Value comparison RS1 and RS2	p-Value comparison RS1 and RS3	p-Value comparison RS2 and RS3	p-Value Friedman or General linear model
**Fundamental Period Measures**				
$F0$	-	-	-	0.454
MCD	-	-	-	0.313^{+}
**Period Perturbation Measures**				
TP	1.000^{+}	0.060^{+}	0.137^{+}	0.026^{++}
$MJit$	0.990^{+}	0.058^{+}	0.290^{+}	0.043^{++}
$Jit(\%)$	1.000^{+}	0.059^{+}	0.136^{+}	0.026^{++}
$JitFac$	1.000^{+}	0.059^{+}	0.135^{+}	0.026^{++}
$JitRat$	1.000^{+}	0.059^{+}	0.136^{+}	0.026^{++}
$PPQ3$	0.843^{+}	0.055^{+}	0.206^{+}	0.028^{++}
$PPQ5$	0.278^{+}	0.042^{+}	0.142^{+}	0.020^{++}
$PPQ11$	-	-	-	0.076^{++}
PPF	1.000^{+}	0.055^{+}	0.133^{+}	0.025^{++}
RAP_B	0.845^{+}	0.055^{+}	0.206^{+}	0.029^{++}
RAP_K	0.845^{+}	0.055^{+}	0.206^{+}	0.029^{++}
PVI	-	-	-	0.063
**Amplitude Perturbation Measures**				
AP	0.003^{+}	0.000^{+}	0.000^{+}	0.000^{++}
$MShim$	0.003^{+}	0.000^{+}	0.000^{+}	0.000^{++}
$Shim(\%)$	0.000^{+}	0.000^{+}	0.000^{+}	0.000^{++}
$APQ3$	0.007	0.000	0.002	0.000
$APQ5$	0.012^{+}	0.000^{+}	0.002^{+}	0.000^{++}
$APQ11$	0.157	0.003	0.000	0.000
APF	0.003^{+}	0.000^{+}	0.000^{+}	0.000^{++}
AVI	0.144^{+}	0.000^{+}	0.000^{+}	0.000^{++}
**Energy Perturbation Measures**				
$EPQ3$	0.059^{+}	0.022^{+}	0.237^{+}	0.014^{++}
$EPQ5$	0.051^{+}	0.032^{+}	0.209^{+}	0.018^{++}
$EPQ11$	-	-	-	0.088^{++}
EPF	0.105^{+}	0.018^{+}	0.138^{+}	0.011^{++}
**Symmetry Measures**				
$PhAI$	-	-	-	0.267
PhA	-	-	-	0.585
$SpSI$	-	-	-	0.861
SpS	-	-	-	0.142
$AmSI$	0.436	0.007	0.017	0.002
AmS	-	-	-	0.549
$DyRSI$	0.325	0.006	0.030	0.002
$DyRS$	-	-	-	0.861
$WaSI$	0.502	0.005	0.014	0.000

+ Data was assumed to be normally distributed ++ Greenhouse-Geisser correction was applied. Green: $p \leq 0.05$, Red: $p > 0.05$, Blue: parameter was mathematically dependent.

Table S2. p-values of all relevant statistical tests performed for males part 2.

Parameter name	p-Value comparison RS1 and RS2	p-Value comparison RS1 and RS3	p-Value comparison RS2 and RS3	p-Value Friedman or General linear model
**Glottal dynamic characteristics**				
OQ	0.001	0.000	0.000	0.000
CQ	0.159^{+}	0.009^{+}	0.003^{+}	0.002^{++}
SQ	-	-	-	0.212
SI	0.159^{+}	0.052^{+}	0.070^{+}	0.017^{++}
RQ	0.024	0.003	0.006	0.000
AQ	0.159^{+}	0.052^{+}	0.070^{+}	0.017^{++}
GGI	0.002	0.011	0.110	0.000
PQ	-	-	-	0.705
GAI	0.000	0.000	0.000	0.000
**Mechanical Measures**				
MADR	0.000^{+}	0.000^{+}	0.000^{+}	0.000^{++}
AmQ	0.000^{+}	0.000^{+}	0.000^{+}	0.000^{++}
Stiff	0.088^{+}	0.001^{+}	0.000^{+}	0.000^{++}
PCV	0.000^{+}	0.000^{+}	0.000^{+}	0.000^{++}
PA	0.000^{+}	0.000^{+}	0.000^{+}	0.000^{++}
ALR	0.000^{+}	0.000^{+}	0.000^{+}	0.000^{++}

+ Data was assumed to be normally distributed ++ Greenhouse-Geisser correction was applied. Green: $p \leq 0.05$, Red: $p > 0.05$, Blue: parameter was mathematically dependent.
