# Supplementary material for: Influence of spatial camera resolution in high-speed videoendoscopy on laryngeal parameters
Source: PLoS One. 2019 Apr 22;14(4):e0215168. doi: 10.1371/journal.pone.0215168 (PMC6476512; doi:10.1371/journal.pone.0215168)
Supplement: S1 Proof — (PDF) [file pone.0215168.s004.pdf]

# S1 Proof

## PPQ5 is less than PPQ3 and PPQ11 under certain conditions

We proof that $PPQ5(\overrightarrow{T}) < PPQ3(\overrightarrow{T})$ and $PPQ5(\overrightarrow{T}) < PPQ11(\overrightarrow{T})$ if every second cycle length $T_i$ is prolonged.

Theorem A: Let $N$ be a non-negative integer and $\overrightarrow{T} \in \mathbb{R}^N$ be a vector of cycle lengths in real numbers, which is component-wise greater than zero and is defined as $\overrightarrow{T} = \{x, x+c, x, x+c, ...\}$ or $\overrightarrow{T} = \{x+c, x, x+c, x...\}$ with $x$ and $c$ greater than zero. Then:

a) $PPQ5(\overrightarrow{T}) < PPQ3(\overrightarrow{T}) \quad \forall \overrightarrow{T} \in \mathbb{R}^N$ and $N = 6, 7, 8, 9, 10, ...$

b) $PPQ5(\overrightarrow{T}) < PPQ11(\overrightarrow{T}) \quad \forall \overrightarrow{T} \in \mathbb{R}^N$ and $N = 12, 13, 14, 15, 16, ...$

Proof:

$$a) \qquad PPQk = \frac{1}{N-k} \sum_{i=\frac{k-1}{2}}^{N-\frac{k-1}{2}-1} \left| 1 - \frac{k \cdot T(i)}{\sum_{j=-\frac{k-1}{2}}^{\frac{k-1}{2}} T(i+j)} \right| \cdot 100$$

$$\Rightarrow \qquad PPQ3 = \frac{1}{N-3} \sum_{i=1}^{N-2} \left| 1 - \frac{3 \cdot T(i)}{T(i-1) + T(i) + T(i+1)} \right| \cdot 100$$

Case distinction: case 1: $N$ is even

$$\begin{aligned} PPQ3 &= \frac{1}{N-3} \cdot \left( \frac{N-2}{2} \left| 1 - \frac{3x}{x+c+x+x+c} \right| + \frac{N-2}{2} \left| 1 - \frac{3x+3c}{x+x+c+x} \right| \right) \cdot 100 \\ &= \frac{1}{N-3} \cdot \left( \frac{N-2}{2} \left| \frac{2c}{3x+2c} \right| + \frac{N-2}{2} \left| \frac{-2c}{3x+c} \right| \right) \cdot 100 \\ &= \frac{N-2}{2N-6} \cdot \left( \frac{2c}{3x+2c} + \frac{2c}{3x+c} \right) \cdot 100 \\ &= \left( \underbrace{\frac{2Nc-4c}{6Nx+4Nc-18x-12c}}_{:=S_1^3} + \underbrace{\frac{2Nc-4c}{6Nx+2Nc-18x-6c}}_{:=S_2^3} \right) \cdot 100 \end{aligned}$$

Analogously for PPQ5

$$\begin{aligned} PPQ5 &= \frac{1}{N-5} \cdot \left( \frac{N-4}{2} \left| 1 - \frac{5x}{x+x+c+x+x+c+x} \right| + \frac{N-4}{2} \left| 1 - \frac{5x+5c}{x+c+x+x+c+x+x+c} \right| \right) \cdot 100 \\ &= \frac{1}{N-5} \cdot \left( \frac{N-4}{2} \left| \frac{2c}{5x+2c} \right| + \frac{N-4}{2} \left| \frac{-2c}{5x+3c} \right| \right) \cdot 100 \\ &= \frac{N-4}{2N-10} \cdot \left( \frac{2c}{5x+2c} + \frac{2c}{5x+3c} \right) \cdot 100 \\ &= \left( \underbrace{\frac{2Nc-8c}{10Nx+4Nc-50x-20c}}_{:=S_1^5} + \underbrace{\frac{2Nc-8c}{10Nx+6Nc-50x-30c}}_{:=S_2^5} \right) \cdot 100 \end{aligned}$$

Now we would like to show that $\frac{S_1^3}{S_2^5} > 1$ and $\frac{S_2^3}{S_1^5} > 1$ for $N$ sufficiently large.

We have:

$$\frac{S_1^3}{S_2^5} = \frac{\frac{2Nc-4c}{6Nx+4Nc-18x-12c}}{\frac{2Nc-8c}{10Nx+6Nc-50x-30c}} = \frac{12N^2c^2 - 84Nc^2 + 120c^2 + 20N^2cx - 140Ncx + 200cx}{8N^2c^2 - 56Nc^2 + 96c^2 + 12N^2cx - 84Ncx + 144cx} := \frac{Q_1(N)}{Q_2(N)} \text{ and}$$
$$\frac{Q_1(6)}{Q_2(6)} = \frac{432c^2 - 504c^2 + 120c^2 + 720cx - 840cx + 200cx}{288c^2 - 336c^2 + 96c^2 + 432cx - 504cx + 144cx} = \frac{48c^2 + 80cx}{48c^2 + 72cx} > 1 \quad \forall x, c > 0$$

Now we investigate $Q_1$ and $Q_2$ as functions of $t \in [6, \infty)$
Obviously $Q_1(6) > Q_2(6)$. In order to show that $Q_1(t) > Q_2(t)$ for all $t \in [6, \infty)$
it is sufficient to show that $Q_1'(t) > Q_2'(t)$ $\forall t \in [6, \infty)$.

We have:

$$\begin{aligned} Q_1'(t) &= 24tc^2 - 84c^2 + 40tcx - 140cx \\ &= 16tc^2 - 56c^2 + \underbrace{8tc^2 - 28c^2}_{\geq 20c^2 \; if \; t \geq 6} + 24tcx - 84cx + \underbrace{16tcx - 56cx}_{\geq 40cx \; if \; t \geq 6} \\ &\geq 16tc^2 - 56c^2 + 24tcx - 84cx \\ &= Q_2'(t) \end{aligned}$$

Furthermore it holds:

$$\frac{S_2^3}{S_1^5} = \frac{\frac{2Nc-4c}{6Nx+2Nc-18x-6c}}{\frac{2Nc-8c}{10Nx+4Nc-50x-20c}} = \frac{8N^2c^2 - 56Nc^2 + 80c^2 + 20N^2cx - 140Ncx + 200cx}{4N^2c^2 - 28Nc^2 + 48c^2 + 12N^2cx - 84Ncx + 144cx} := \frac{Q_3(N)}{Q_4(N)} \text{ and}$$
$$\frac{Q_3(6)}{Q_4(6)} = \frac{288c^2 - 336c^2 + 80c^2 + 720cx - 840cx + 200cx}{144c^2 - 168c^2 + 48c^2 + 432cx - 504cx + 144cx} = \frac{32c^2 + 80cx}{24c^2 + 72cx} > 1 \quad \forall x, c > 0$$

Now we investigate $Q_3$ and $Q_4$ as functions of $t \in [6, \infty)$
Obviously $Q_3(6) > Q_4(6)$. In order to show that $Q_3(t) > Q_4(t)$ for all $t \in [6, \infty)$
it is sufficient to show that $Q_3'(t) > Q_4'(t)$ $\forall t \in [6, \infty)$.

We have:

$$\begin{aligned} Q_3'(t) &= 16tc^2 - 56c^2 + 40tcx - 140cx \\ &= 8tc^2 - 28c^2 + \underbrace{8tc^2 - 28c^2}_{\geq 20c^2 \; if \; t \geq 6} + 24tcx - 84cx + \underbrace{16tcx - 56cx}_{\geq 40cx \; if \; t \geq 6} \\ &\geq 8tc^2 - 28c^2 + 24tcx - 84cx \\ &= Q_4'(t) \end{aligned}$$

In summary both summands of PPQ3 are pairwise greater than the summands of PPQ5

Thus PPQ3 is grater than PPQ5 for all x and c $>0$ and all N $\geq 6$ if $N$ is even

Case 2: $N$ is odd

Assume first that: $\overrightarrow{T}=(x,x+c,x,...)$

$$\begin{aligned}
PPQ3 &= \frac{1}{N-3}\cdot\left(\frac{N-3}{2}\left|\frac{2c}{3x+2c}\right|+\frac{N-1}{2}\left|\frac{-2c}{3x+c}\right|\right)\cdot 100\\
&= \left(\frac{N-3}{2\cdot(N-3)}\cdot\frac{2c}{3x+2c}+\frac{N-1}{2N-6}\cdot\frac{2c}{3x+c}\right)\cdot 100\\
&= \left(\underbrace{0.5\cdot\frac{2c}{3x+2c}}_{:=U_1^3}+\underbrace{\frac{2Nc-2c}{6Nx+2Nc-18x-6c}}_{:=U_2^3}\right)\cdot 100\\
PPQ5 &= \frac{1}{N-5}\cdot\left(\frac{N-3}{2}\left|\frac{2c}{5x+2c}\right|+\frac{N-5}{2}\left|\frac{-2c}{5x+3c}\right|\right)\cdot 100\\
&= \left(\frac{N-3}{2N-10}\cdot\frac{2c}{5x+2c}+\frac{N-5}{2\cdot(N-5)}\cdot\frac{2c}{5x+3c}\right)\cdot 100\\
&= \left(\underbrace{\frac{2Nc-6c}{10Nx+4Nc-50x-20c}}_{:=U_1^5}+\underbrace{0.5\cdot\frac{2c}{5x+3c}}_{:=U_2^5<U_1^3}\right)\cdot 100
\end{aligned}$$

Now we would like to show that $\frac{U_2^3}{U_1^5}>1$ for $N$ sufficiently large.

We have:

$$\frac{U_2^3}{U_1^5}=\frac{\frac{2Nc-2c}{6Nx+2Nc-18x-6c}}{\frac{2Nc-6c}{10Nx+4Nc-50x-20c}}=\frac{8N^2c^2-48Nc^2+40c^2+20N^2cx-120Ncx+100cx}{4N^2c^2-24Nc^2+36c^2+12N^2cx-72Ncx+108cx}:=\frac{W_1(N)}{W_2(N)} \text{ and}$$

$$\frac{W_1(7)}{W_2(7)}=\frac{392c^2-336c^2+40c^2+980cx-840cx+100cx}{196c^2-168c^2+36c^2+588cx-504cx+108cx}=\frac{96c^2+240cx}{64c^2+192cx}>1\quad\forall x,c>0$$

Now we investigate $W_1$ and $W_2$ as functions of $t\in[7,\infty)$

Obviously $W_1(7)>W_2(7)$. In order to show that $W_1(t)>W_2(t)$ for all $t\in[7,\infty)$

it is sufficient to show that $W_1'(t)>W_2'(t)\ \forall t\in[7,\infty)$.

We have:

$$\begin{aligned}
W_1'(t) &= 16tc^2-48c^2+40tcx-120cx\\
&= 8tc^2-24c^2+\underbrace{8tc^2-24c^2}_{\geq 32c^2\ if\ t\geq 7}+24tcx-72cx+\underbrace{16tcx-48cx}_{\geq 64cx\ if\ t\geq 7}\\
&\geq 8tc^2-24c^2+24tcx-72cx\\
&= W_2'(t)
\end{aligned}$$

In summary both summands of PPQ3 are pairwise greater than the summands of PPQ5
Thus PPQ3 is grater than PPQ5 for all x and c > 0 and all N ≥ 7 if $N$ is odd and $\overrightarrow{T} = (x, x+c, x, ...)$
Finally the case N odd, $\overrightarrow{T} = (x+c, x, x+c, ...)$ is fully analogous.

b) analogously for PPQ11 if $N$ is even

$$\begin{aligned} PPQ11 &= \frac{1}{N-11} \cdot \left( \frac{N-10}{2} \left| 1 - \frac{11x}{11x+6c} \right| + \frac{N-10}{2} \left| 1 - \frac{11x+11c}{11x+5c} \right| \right) \cdot 100 \\ &= \frac{1}{N-11} \cdot \left( \frac{N-10}{2} \left| \frac{6c}{11x+6c} \right| + \frac{N-10}{2} \left| \frac{-6c}{11x+5c} \right| \right) \cdot 100 \\ &= \frac{N-10}{2N-22} \cdot \left( \frac{6c}{11x+6c} + \frac{6c}{11x+5c} \right) \cdot 100 \\ &= \frac{N-10}{2N-22} \cdot \left( \frac{2c}{3.\bar{6}x+2c} + \frac{2c}{3.\bar{6}x+1.\bar{6}c} \right) \cdot 100 \end{aligned}$$

Now from $\frac{N-10}{2N-22} > \frac{N-4}{2N-10} \quad \forall N \geq 12$

and $\left( \frac{2c}{3.\bar{6}x+2c} + \frac{2c}{3.\bar{6}x+1.\bar{6}c} \right) > \left( \frac{2c}{5x+2c} + \frac{2c}{5x+3c} \right) \quad \forall x, c > 0$

we conclude that PPQ11 is grater than PPQ5 for all x and c > 0 and all N ≥ 12 if $N$ is even.

If the number of elements in $\overrightarrow{T}$ is odd and $\overrightarrow{T} = (x, x+c, x, ...)$

$$\begin{aligned} PPQ11 &= \frac{1}{N-11} \cdot \left( \frac{N-11}{2} \left| \frac{6c}{11x+6c} \right| + \frac{N-9}{2} \left| \frac{-6c}{11x+5c} \right| \right) \cdot 100 \\ &= \left( \frac{N-11}{2 \cdot (N-11)} \cdot \frac{6c}{11x+6c} + \frac{N-9}{2N-22} \cdot \frac{6c}{11x+5c} \right) \cdot 100 \\ &= \left( \underbrace{0.5 \cdot \frac{2c}{3.\bar{6}x+2c}}_{:=U_1^{11} > U_2^5} + \frac{N-9}{2N-22} \cdot \frac{2c}{3.\bar{6}x+1.\bar{6}c} \right) \cdot 100 \end{aligned}$$

Now from $\frac{N-9}{2N-22} > \frac{N-3}{2N-10} \quad \forall N \geq 13$

and $\frac{2c}{3.\bar{6}x+1.\bar{6}c} > \frac{2c}{5x+2c} \quad \forall x, c > 0$

we conclude that PPQ11 is grater than PPQ5 for all x and c > 0 and all N ≥ 13 if $N$ is odd and $\overrightarrow{T} = (x, x+c, x, ...)$.
The case N odd, $\overrightarrow{T} = (x+c, x, x+c, ...)$ is again fully analogous.

Summary: PPQ11 is grater than PPQ5 for all x and c > 0 and all N ≥ 13 if $N$ is odd.
Therefore PPQ3 and PPQ11 are greater than PPQ5 if every second cycle length $T_i$ is prolonged for even and odd numbers of elements in $\overrightarrow{T}$ and sufficiently large N. This analogously applies to all APQ and EPQ measures.
