# Supplementary material for: Influence of spatial camera resolution in high-speed videoendoscopy on laryngeal parameters
Source: PLoS One. 2019 Apr 22;14(4):e0215168. doi: 10.1371/journal.pone.0215168 (PMC6476512; doi:10.1371/journal.pone.0215168)
Supplement: S2 Proof — (PDF) [file pone.0215168.s005.pdf]

S2 Appendix

PVI and AVI are independent of the order of their elements

We proof that PVI ($\vec{T}$) and AVI($\vec{A}$) are independent of the order of the elements within $\vec{T}$ or $\vec{A}$.

THEOREM B: Let N be an integer ≥ 2, let $\vec{A}$ and $\vec{T}$ be vectors in $\mathbb{R}^N$ in real numbers, which are component-wise greater than zero. Then:

a) $PVI(T_0, ..., T_{N-1}) = PVI(T_{p(0)}, ..., T_{p(N-1)})$ for any bijective $p : \{0, ..., N-1\} \rightarrow \{0, ..., N-1\}$

b) $AVI(A_0, ..., A_{N-1}) = AVI(A_{p(0)}, ..., A_{p(N-1)})$ for any bijective $p : \{0, ..., N-1\} \rightarrow \{0, ..., N-1\}$

Proof:

$a)$ we define:

$$\bar{T} = \frac{1}{N} \sum_{j=0}^{N-1} T_j \quad , \quad T_{sum} = \sum_{i=0}^{N-1} \left| T_i - \bar{T} \right|^2$$

and observe:

$$\begin{aligned} PVI(\vec{T}) &= 1000 \cdot \frac{\frac{1}{N} \sum_{i=0}^{N-1} \left| T_i - \frac{1}{N} \sum_{j=0}^{N-1} T_j \right|^2}{\left(\frac{1}{N} \sum_{j=0}^{N-1} T_j \right)^2} \\ &= 1000 \cdot \frac{\frac{1}{N} \sum_{i=0}^{N-1} \left| T_i - \bar{T} \right|^2}{\left(\bar{T} \right)^2} \\ &= 1000 \cdot \frac{\frac{1}{N} T_{sum}}{\left(\bar{T} \right)^2} \end{aligned}$$

As neighter T_{sum} nor $\bar{T}$ depend on the order of $\vec{T}$ this is also true for PVI.

$b)$ $AVI(\vec{A}) = log_{10} \left(PVI(\vec{A}) \right)$.

Thus AVI ($\vec{A}$) is independent of the order of the elements in $\vec{A}$ as well.
