# Supplementary material for: Influence of spatial camera resolution in high-speed videoendoscopy on laryngeal parameters
Source: PLoS One. 2019 Apr 22;14(4):e0215168. doi: 10.1371/journal.pone.0215168 (PMC6476512; doi:10.1371/journal.pone.0215168)
Supplement: S3 Proof — (PDF) [file pone.0215168.s006.pdf]

S3 Appendix

Similar behavior of MShim and APF for low perturbation

We proof that the ratio $\frac{APF(\vec{A})}{MShim(\vec{A})}$ approaches $5 \cdot \ln 10 = 11.5129...$ if the amplitude perturbation becomes infinitely small.

Theorem C: Let N be a non-negative integer ≥ 2, let $\vec{A} \in \mathbb{R}^N$ be a vector of amplitudes in real numbers, which is component-wise greater than zero and let $\frac{A_i}{A_{i+1}} \to 1 \; \forall i = 0, ..., N-1$. then: $\frac{APF(\vec{A})}{MShim(\vec{A})} \to 5 \cdot \ln 10$

Proof:

We have:

$$MShim(\vec{A}) = \frac{20}{N-1} \sum_{i=0}^{N-2} \left| \log_{10} \left[\frac{A_i}{A_{i+1}} \right] \right|$$

$$APF(\vec{A}) = \frac{1}{N-1} \sum_{i=1}^{N-1} \left| \frac{A_i - A_{i-1}}{A_i} \right| \cdot 100 \; = \frac{20}{N-1} \sum_{i=0}^{N-2} \left| \frac{A_{i+1} - A_i}{A_{i+1}} \right| \cdot 5 \; = \frac{20}{N-1} \sum_{i=0}^{N-2} \left| 5 \cdot \left(1 - \frac{A_i}{A_{i+1}} \right) \right|$$

Now we assume: $\frac{A_i}{A_{i+1}} \to 1 \quad \forall i \in \{0, 1, 2, ..., N-1\}$

We have: $$\frac{APF(\vec{A})}{MShim(\vec{A})} = \frac{\frac{20}{N-1} \sum_{i=0}^{N-2} \left| 5 \cdot \left(1 - \frac{A_i}{A_{i+1}} \right) \right|}{\frac{20}{N-1} \sum_{i=0}^{N-2} \left| \log_{10} \left[\frac{A_i}{A_{i+1}} \right] \right|}$$

Substituting: $x_0 = \frac{A_0}{A_1}, x_1 = \frac{A_1}{A_2}, ..., x_{N-2} = \frac{A_{N-2}}{A_{N-1}}$

The right hand side for $\frac{APF(\vec{A})}{MShim(\vec{A})}$ becomes:

$$\frac{\frac{20}{N-1} \sum_{i=0}^{N-2} |5 \cdot (1 - x_i)|}{\frac{20}{N-1} \sum_{i=0}^{N-2} |\log_{10} x_i|} \qquad (*)$$

As this is a continuos function of $(x_0, ..., x_{N-2})$ we can derive the limit of $(*)$ for $(x_0, ..., x_{N-2}) \to (1, ..., 1)$ using a sequence of type $(x_0, ..., x_{N-2}) \to 1$. Thus $(*)$ in the limit becomes:

$$\lim_{x \to 1} \frac{|5 \cdot (1 - x)|}{|\log_{10}(x)|}$$

Now we consider a single term:

Case: x > 1

$$\begin{aligned}
&\lim_{x\to 1} \frac{|5\cdot(1-x)|}{|\log_{10}(x)|} \\
&= \lim_{x\to 1} \frac{-5\cdot(1-x)}{\log_{10}(x)} && \text{| L'Hôpital} \\
&= \lim_{x\to 1} 5\ln(10)\cdot x \\
&= 5\ln(10)
\end{aligned}$$

Case: x < 1

$$\begin{aligned}
&\lim_{x\to 1} \frac{|5\cdot(1-x)|}{|\log_{10}(x)|} \\
&= \lim_{x\to 1} \frac{5\cdot(1-x)}{-\log_{10}(x)} && \text{| L'Hôpital} \\
&= \lim_{x\to 1} 5\ln(10)\cdot x \\
&= 5\ln(10)
\end{aligned}$$

Therefore, as shown above, if the amplitude perturbation becomes small APF is about 11.513 times MShim
