# Supplementary figures and images for: Influence of spatial camera resolution in high-speed videoendoscopy on laryngeal parameters
Source: PLoS One. 2019 Apr 22;14(4):e0215168. doi: 10.1371/journal.pone.0215168 (PMC6476512; doi:10.1371/journal.pone.0215168)

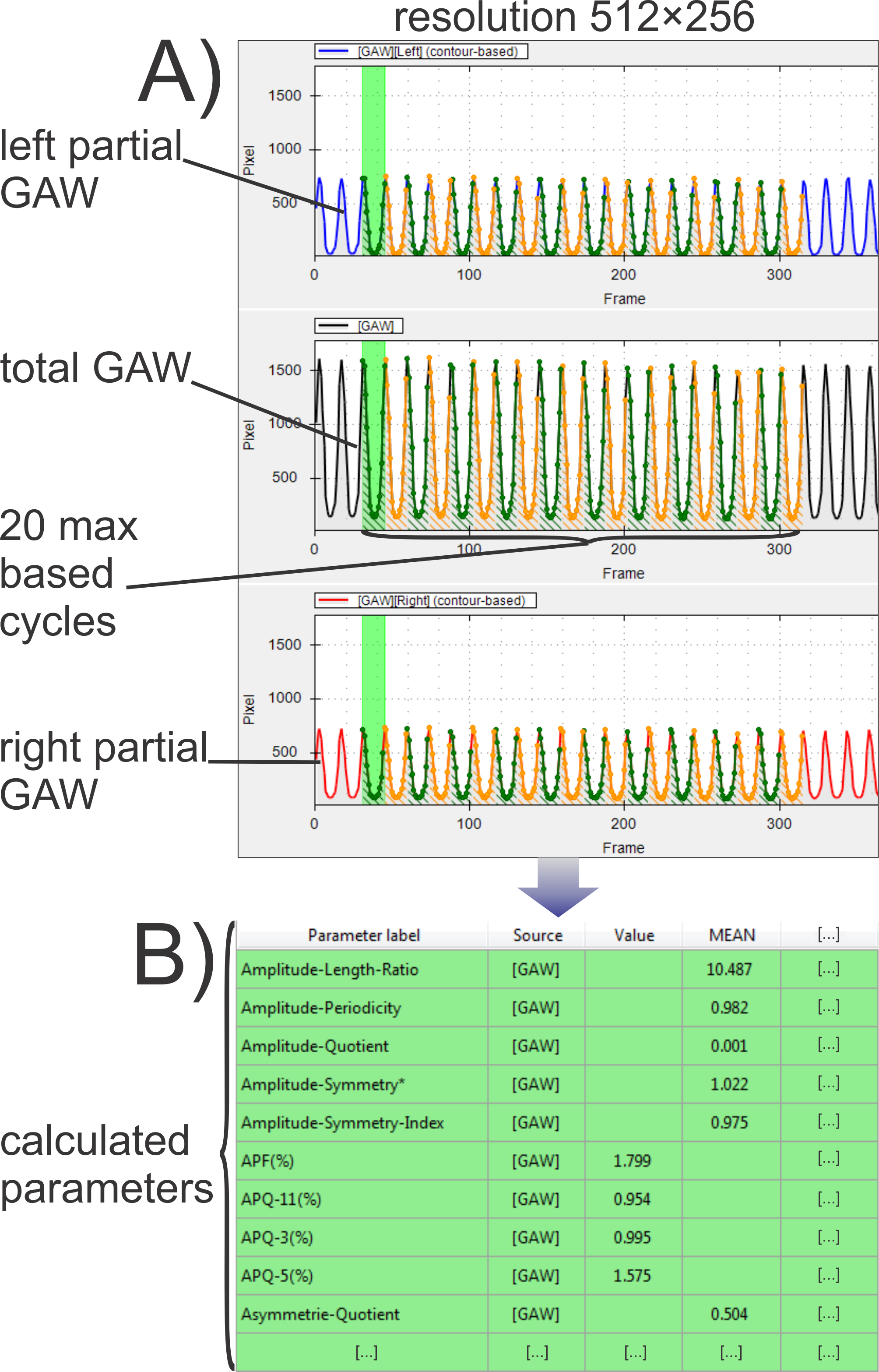

Supplement: S1 Fig — A) Detection of 20 max based cycles (each cycle starts at a local maximum and ends before the next local maximum) in total and partial GAWs and B) Calculation of in total 50 different cycle based parameters. (TIF) [file pone.0215168.s007.tif]

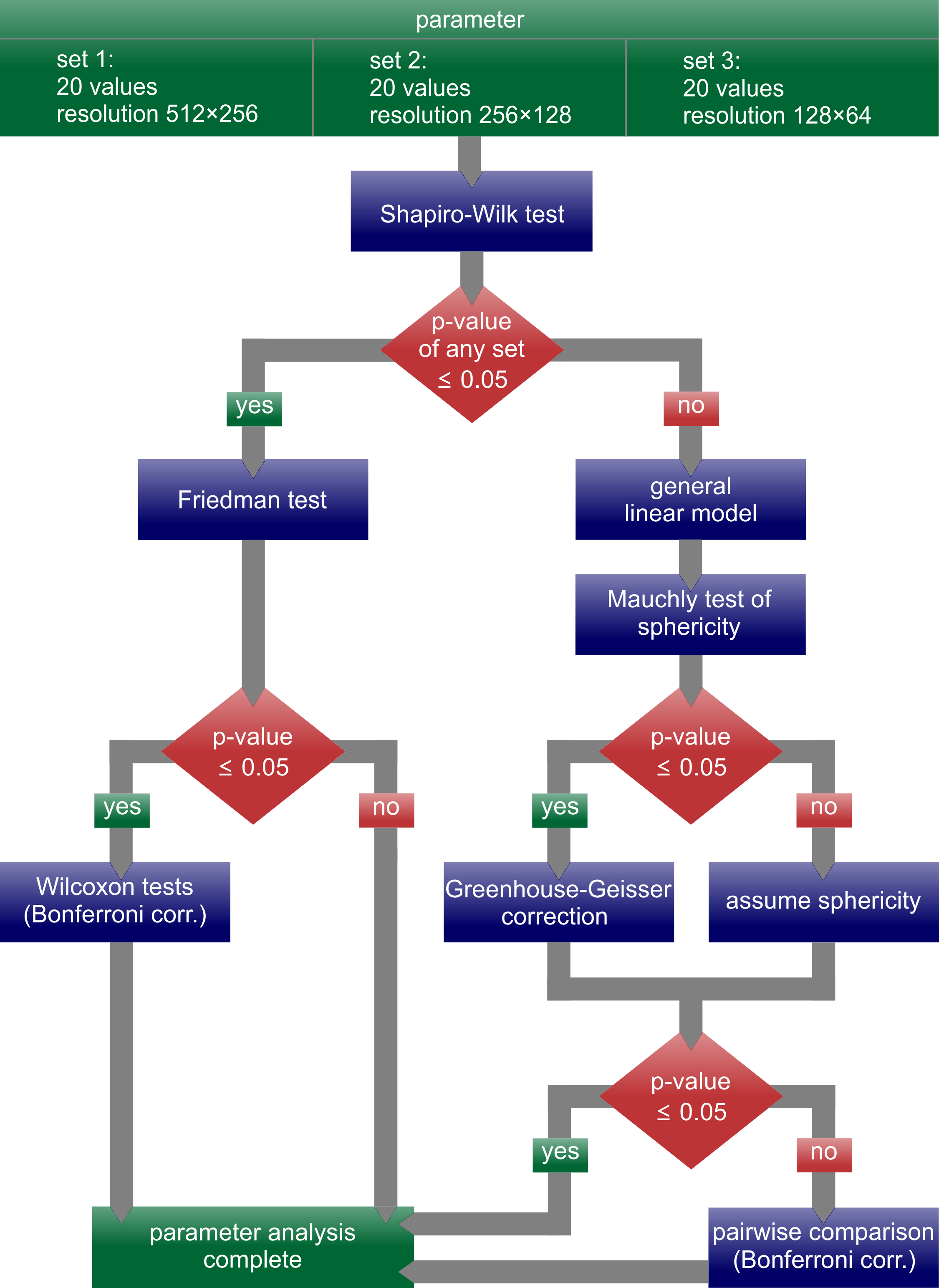

Supplement: S2 Fig — For each parameter three sets from recordings with different resolutions of 20 values each were calculated. For each set of three sets, the illustrated statistical analysis workflow was performed. (TIF) [file pone.0215168.s008.tif]
